# Systematic analysis of the expression and prognosis relevance of FBXO family reveals the significance of FBXO1 in human breast cancer

**DOI:** 10.1186/s12935-021-01833-y

**Published:** 2021-02-23

**Authors:** Yaqian Liu, Bo Pan, Weikun Qu, Yilong Cao, Jun Li, Haidong Zhao

**Affiliations:** 1 Department of Oncology & Department of Breast Surgery, The Second Hospital of Dalian Medical University, Dalian, 116023 China; 2 Department of Hepatobiliary and Pancreatic Surgery, The Second Hospital of Dalian Medical University, Dalian, 116023 China

**Keywords:** F-box protein, Biomarkers, Prognosis, Breast cancer, Bioinformatics analysis

## Abstract

**Background:**

Breast cancer (BC) remains a prevalent and common form of cancer with high heterogeneity. Making efforts to explore novel molecular biomarkers and serve as potential disease indicators, which is essential to effectively enhance the prognosis and individualized treatment of BC. FBXO proteins act as the core component of E3 ubiquitin ligase, which play essential regulators roles in multiple cellular processes. Recently, research has indicated that FBXOs also play significant roles in cancer development. However, the molecular functions of these family members in BC have not been fully elucidated.

**Methods:**

In this research, we investigated the expression data, survival relevance and mutation situation of 10 FBXO members (FBXO1, 2, 5, 6, 16, 17, 22, 28, 31 and 45) in patients with BC from the Oncomine, GEPIA, HPA, Kaplan–Meier Plotter, UALCAN and cBioPortal databases. The high transcriptional levels of FBXO1 in different subtypes of BC were verified by immunohistochemical staining and the specific mutations of FBXO1 were obtained from COSMIC database. Top 10 genes with the highest correlation to FBXO1 were identified through cBioPortal and COXPRESdb tools. Additionally, functional enrichment analysis, PPI network and survival relevance of FBXO1 and co-expressed genes in BC were obtained from DAVID, STRING, UCSC Xena, GEPIA, bc-GenExMiner and Kaplan–Meier Plotter databases. FBXO1 siRNAs were transfected into MCF-7 and MDA-MB-231 cell lines. Expression of FBXO1 in BC cell lines was detected by western-blot and RT-qPCR. Cell proliferation was detected by using CCK-8 kit and colony formation assay. Cell migration was detected by wound‐healing and transwell migration assay.

**Results:**

We found that FBXO2, FBXO6, FBXO16 and FBXO17 were potential favorable prognostic factors for BC. FBXO1, FBXO5, FBXO22, FBXO28, FBXO31 and FBXO45 may be the independent poor prognostic factors for BC. All of them were correlated to clinicopathological staging. Moreover, knockdown of FBXO1 in MCF7 and MDA-MB-231 cell lines resulted in decreased cell proliferation and migration in vitro. We identified that FBXO1 was an excellent molecular biomarker and therapeutic target for different molecular typing of BC.

**Conclusion:**

This study implies that FBXO1, FBXO2, FBXO5, FBXO6, FBXO16, FBXO17, FBXO22, FBXO28, FBXO31 and FBXO45 genes are potential clinical targets and prognostic biomarkers for patients with different molecular typing of BC. In addition, the overexpression of FBXO1 is always found in breast cancer and predicts disadvantageous prognosis, implicating it could as an appealing therapeutic target for breast cancer patients.

## Background

Breast cancer (BC) is among the most common malignant tumor (11.6%) and the leading cause of cancer death (6.6%) globally in women [1]. Classical clinical prognostic biomarkers such as estrogen receptor (ER), progesterone receptor (PR), and human epidermal growth factor receptor 2 (HER2) have played crucial roles in determination of which patients may benefit from target therapy or endocrine treatment [2]. However, considering the heterogeneity of tumor and individual differences in patients, the existing biomarkers have some limitations in predicting the prognosis of BC. Hence, there is an urgent need to explore novel molecular biomarkers as prognostic indicators in the field of clinical research, which perhaps contribute to improve the prognosis and guide the individualized treatment strategies for BC patients.

Ubiquitin proteasome pathway is the most important protein degradation pathway with high selectivity in human, which plays a critical role in tumorigenesis and pathological mechanism of tumor. In ubiquitination cascade pathway, E3 Ubiquitin ligase is known as the second prevalent tumor‐related functional gene family after protein kinases, which is a novel anticancer drug target, for its specific recognition of target protein by proteasome [3]. F-box proteins are the core component of the SKP1-cullin 1-F-box (SCF)-type E3 ubiquitin ligase, which can be classified into three sub-families: (1) F-box with leucine rich amino acid repeats (FBXL); (2) F-box with WD 40 amino acid repeats (FBXW); (3) F-box only with uncharacterized domains (FBXO) [3]. Generally, F-box proteins act as molecular regulators in multiple biological processes of cell like cell cycle, epithelial-mesenchymal transition (EMT), cell apoptosis and many signaling pathways related to tumor such as P13K-AKT-mTOR, p53 and NRF2 [4, 5]. F-box proteins directly bind to substrates which modified by proper post-translational modification, and mediate ubiquitination and subsequent degradation of the target protein [5]. As the largest sub-family of F-box proteins, FBXO has 37 members, it has been verified that many of them are closely related to tumor biological processes according to many studies.

FBXO1, also known as cyclin F (CCNF), mainly contains a cyclin box domain. The main function of FBXO1 is participating in centrosome duplication and DNA repair through SCF-type E3 ligase [6]. It participates in regulation of various cell cycle-related processes including DNA replication and repair, centrosome duplication, maintenance of genome stability [7]. FBXO2 (Fbs1 or FBG1) functions as a component of the S phase kinase-associated protein 1-cullin 1-F-box protein (SKP1-CUL1-SCF) ubiquitin ligase complex, which tends to distribute in human brain related to nervous or psychical diseases. The specific substrates of FBXO2 are high-mannose type asparagine (N)-linked glycoprotein [8]. FBXO5 (Emi1/FBX5) has been suggested to play crucial roles in the development of HCC, cervical cancer and squamous-cell lung carcinoma in the latest research on bioinformatics analysis [9–11]. It has been proved that FBXO5 connects with the anaphase promoting complex/cyclosome (APC/C) co-activator proteins to inhibit APC/C activation, and stabilizes ubiquitin substrates which has oncogenic activity to govern cell cycle progression to S phase and mitosis [12]. Impaired expression of FBXO6 (also called FBG2) increases the therapeutic resistance in cancer cells by inducing the degradation of target molecules in ubiquitin‐mediated cellular pathways. Zhang et al*.* [13] have found that FBXO6 facilitates the ubiquitination and mediates the degradation of Chk1 to increase certain drugs resistance of tumor cells. As the component of the SCF complex, FBXO16 interacts physically with the C-terminal domain of nuclear β-catenin protein to promote its lysine 48-linked polyubiquitination and mediate degradation of β-catenin [14]. It inhibits EMT by attenuating the levels of β-catenin. The main function of FBXO17 is targeting glycogen synthase kinase-3β to the E3 ubiquitin ligase protein complex for polyubiquitination and proteasomal degradation [15]. Recent studies have showed that overexpression of FBXO17 increases cell proliferation coupled with Akt activation in lung adenocarcinoma [16]. FBXO22 is a hemedependent binding protein to Bach1, which is also a pro‐metastatic transcription factor [17]. In BC, FBXO22 determines the sensitivity of endocrine treatment by making KDM4B ubiquitination complexed with unliganded or selective estrogen receptor modulators (SERMs)‐bound estrogen receptor (ER) [18]. FBXO28 regulates topoisomerase IIα decatenation activity and plays an important role in maintaining cell genomic stability [19]. It has been reported that FBXO28 may have a carcinogenic effect through non-proteolytic ubiquitination of MYC143 to stimulate transcription in BC [20]. FBXO31, also known as FBXO14, have been showed as a tumor suppressor protein. It targets and ubiquitylates slug for proteasomal degradation. Due to its growth-suppression activity, it is downregulated in many kinds of cancers [21]. FBXO45 is an evolutionary conserved F-box protein, it contains a conserved F-box domain and a SPRY domain, which recruits alternate RING-finger protein substrates to the ubiquitin ligase complex [22]. There is an evidence that FBXO45 can target p73 in vitro and in vivo to regulate the apoptosis mediated by p53 in tumor cells [23].

Although the functions of FBXO family members have been studied in some researches as mentioned above, the expression profiles of important FBXO family members in BC and the relationship between expression of FBXO genes and prognosis of BC are still worth exploring. In this article, we firstly evaluated the expression levels, mutation situations and prognosis relevance of the 10 important FBXO family members (FBXO1, 2, 5, 6, 16, 17, 22, 28, 31 and 45), which have intimate connection with BC. Therefore, we screened out FBXO1, which is overexpressed in BC and significantly correlated with the prognosis of BC patients. To further analyze the cellular function of FBXO1, we have successfully established the FBXO1-knockdown breast cancer cell lines and explore the effect of FBXO1 on cell function. Next, we screened out the functional gene cluster of FBXO1 and constructed the Protein–Protein Interaction (PPI) network by analyzing large datasets available in various public databases.

## Material and methods

### Oncomine database

The human mRNA expression levels of FBXO gene family members in BC were compared with normal tissues by using the Oncomine gene expression array database (http://www.oncomine.org), an integrated data-mining platform. Students’ t test was adopted and transcriptional data of FBXOs were represented as log2-transformed form. We conducted the selection criteria as follows: Statistically significant P-values threshold < 1E-2, fold change > 2 and the gene rank in the top 10%. All statistical methods and data source were acquired directly from the online database.

### The gene expression profiling interactive analysis (GEPIA) dataset

The transcriptional levels of FBXOs in breast invasive carcinoma (BRCA) and normal breast tissue were obtained from the GEPIA database (http://gepia.cancer-pku.cn), and a public dataset assembles varieties of gene expression profiling functional modules, which was developed by scientists of Peking University [24]. We focused on the analytical results among intrinsic subtypes of BRCA and normal tissue. The correlation analysis of FBXO1 and related genes in BRCA tumor and normal tissue datasets was based on the GTEx and TCGA data. By using one-way ANOVA test, we defined the absolute value of Log2(FC) cutoff is 1; statistically significant p-value Cutoff is 1E-3. The linear dependence (correlation) between FBXO1 and hub genes was measured using Spearman's correlation coefficient. The results were used the non-log scale for calculation and used the log-scale axis for visualization.

### UALCAN database

UALCAN database (http://ualcan.path.uab.edu/) is a publicly accessible dataset for analyzing 31 cancer types’ OMICS data, which is built on PERL-CGI with high quality graphics using JavaScript and CSS. These resources allow researchers to understand the impact of gene expression levels and gather relative clinicopathological parameters of various individual cancer types from The Cancer Genome Atlas (TCGA) [25]. We acquired the FBXOs’ transcriptional data from TCGA pan-cancer view and major subclasses and stages of BRCA by using UALCAN database. The mRNA information was unified as transcripts per million (TPM) reads for data comparison from different sources. P-value < 0.05 was considered statistically significant.

### The Human Protein Atlas (HPA) database

The Human Protein Atlas (HPA) (https://www.proteinatlas.org) aims to provide 24,000 kinds of human protein distribution information in different tissues and cells, and it displays for more than 20 kind of cancer types’ immunohistochemical staining results. In this work, for comparing the expression difference of the FBXO protein, we showed the immunohistochemical staining images between breast tumor and normal tissues from the HPA database to observe the tissue location of the target protein directly.

### TCGA dataset and cBioPortal online tools

cBioPortal for cancer genomics is an open source resource for interactive exploration of multiple cancer genomic datasets. It allows researchers to visualize and analyze multidimensional genetic changes in different samples, genes and pathways [26]. The Breast Invasive Carcinoma of the cancer genome atlas (TCGA, Firehose Legacy, 1108 total samples) was selected for genomics analysis. By using the cBioPortal online tool (http://www.cbioportal.org), we investigated FBXO gene family’s predicted copy number alterations, mRNA expression (RNA sequencing [RNA-seq] version (v.)2 RSEM), gene correlations and Mutations situation, the results were automatically calculated using a Z-score ± 2.0, Pearson’s correction was considered.

### Bc-GenExMiner (v4.4) online tool

Breast Cancer Gene-Expression Miner (bc-GenExMiner v4.4) online tool (http://bcgenex.centregauducheau.fr/BC-GEM/GEM-Accueil.php) is a statistical mining tool of published BC transcriptomic data (DNA microarrays [n = 10001] and RNA-seq [n = 4712]). It incorporates three classical mining functions: correlation, expression and prognosis [27]. According to common clinical parameters, we analyzed the FBXO gene family’s expression data in different patient groups. The subtypes of parameter include age, nodal status, ER, PR, HER-2, Basal-like statues, Triple-negative statues (IHC) and P53 status (sequence-based). Scarff-Bloom-Richardson (SBR) grade, and Nottingham prognostic index (NPI). The correlative heatmap of FBXO1 and the cell cycle pathway related hub genes was drawn by using the correlation module.

### Kaplan–Meier plotter (KM plotter) database for survival analysis

We evaluated the prognostic significance of FBXO family members in KM plotter online database (http://kmplot.com/). The KM plotter was utilized to estimate the effect of 54 k genes (mRNA, miRNA, protein) on survival in 21 cancer types based on the gene arrays, RNA-sequence or next generation sequencing (for mutation data). Sources for the databases include GEO, EGA, and TCGA. The correlation between the target gene mRNA expression levels and disease-free survival rate (DFS), the overall survival (OS) rate, distance metastasis free survival (DMFS) and post progression survival (PPS) in BC groups were calculated by the Kaplan–Meier curve and log-rank test. The results were shown in the Kaplan–Meier survival plots. Hazard ratio (HR) and 95% confidence were calculated automatically by website tool. The values of each group are shown as the mean ± SD. P-value < 0.05 was regarded as statistically significant by using Log-rank test.

### University of California Santa Cruz (UCSC) cancer genomics browser

UCSC Xena functional genomic browser is a database maintained by the University of California, Santa Cruz (UCSC). It is a new generation of online data analysis and visualization platform integrating analysis, visualization and galaxy. This tool contains the common standardized the data from TCGA, ICGC, TARGET, GTEX and CCLE datasets [28]. We used the UCSC Xena browser (http://xena.ucsc.edu/) to explore the correlation between FBXO1 and co-expression genes expression in different BC subtypes. The result of the comparison was evaluated by Spearman's correlation and represented in heat-map form.

### Catalogue of Somatic Mutations in Cancer (COSMIC) database

COSMIC is the world's largest and most comprehensive resource for exploring the impact of somatic mutations in human cancer (https://cancer.sanger.ac.uk/cosmic). It includes somatic mutation data from different research institutions and databases, and provides convenient browsing, retrieval and downloading functions. The main goal is to conduct in-depth study on cell samples commonly used in cancer research and analyze their mutation information [29]. We used the pie charts to depict the mutations in FBXO1 in BC and the distribution and substitutions on the coding strand.

### Functional enrichment analysis

COXPRESdb is a comprehensive dataset that comparing coexpression-gene in seven model animals (https://coxpresdb.jp/) [30]. We used cBioPortal database and COXPRESdb dataset to screen out the top human 150 genes with the strongest correlation with FBXO1, and obtained the intersect genes from both of databases. The functions of FBXO1 and the genes significantly associated with FBXO1 were predicted by gene ontology (GO) and Kyoto Encyclopedia of Genes and Genomes (KEGG) pathway analysis. GO enrichment includes biological process (BP), cell component (CC), molecular function (MF). By referring to STRING database (https://string-db.org/), we screened the items with corrected P value ≤ 0.05. A total of 313 biological processes, 36 molecular functions and 56 cell components are related. Using R 3.6.3 software, we installed clusterProfiler, enrichplot and ggplot2 package to draw the histogram and bubble chart of the most remarkable results of GO and KEGG enrichment analysis. Using the Database for Annotation, Visualization and Integrated Discovery (DAVID) database (http://david.abcc.ncifcrf.gov/), we annotated the key targets in hsa04110 via Fisher's exact test: Cell cycle pathway to reveal the possible pathogenesis mediated by critical genes in breast adenocarcinoma.

### Protein–protein interactions (PPI) network analysis

The PPI of co-expressed genes was retrieved from STRING database with an interaction score > 0.4, and we reconstructed the data via Cytoscape software (version 3.6.1) [31]. Molecular Complex Detection (MCODE) plug-in was employed to locate the densest connected module to find hub genes of clusters based on topology. The parameter standard as follows: MCODE score > 5 points, degree cut-off is 2, node score cut-off is 0.2, Max depth is 100, and k-Score is 2. The top 10 hub genes were verified according to the degree-rank by CytoHubba plug-in. Next, we analyzed the potential biological process of hub genes by using BINGO plug-in. We selected hypergeometic text and Benjamin & Hochberg False Discovery Correction (FDR) method. The significance P-value set to 0.05.

### Immunohistochemistry (IHC)

The IHC analysis was conducted to evaluate the expression of FBXO1 in different clinical molecular subtypes of BC tissues. In brief, following 4% formalin fixation and paraffin-embedding of specimens, 3 μm thick sections were incubated with primary Rabbit anti-FBXO1 antibody (1:200, Sigma, Louis, MO, USA) overnight at 4 °C, washed 3 times with PBS, and incubated with the secondary antibody for 1 h at 37 °C and streptavidin-HRP. The DAB kit was purchased from Zhongshan Goldenbridge Biotechnology Company (Beijing, China). The sections were stained with hematoxylin. The breast tumor specimens of patients were obtained from department of pathology, the Second Affiliated Hospital of Dalian Medical University (Dalian, China). The research protocol was approved and recorded by the Ethics Committee of The Second Affiliated Hospital of Dalian Medical University. All procedures are carried out in accordance with the Helsinki Declaration.

### Cell culture and small interfering RNA-mediated silencing

All human breast cell lines (MCF-10A, MCF7, MDA-MB-231, MDA-MB-468, SK-BR3, T47D, HCC1954 and BT474) were obtained from The American Type Culture Collection (ATCC, Manassas, VA, USA) and cultured in 1640 and DMEM media respectively containing 10% fetal bovine serum (Gibco, Carlsbad, CA, USA) and penicillin/streptomycin (Hyclone, Logan, Utah, USA). Cells were maintained in a 5% CO_2_ humidified incubator at 37 °C. FAM fluorescence labeled gene‐specific oligonucleotides and negative control oligonucleotides (GenePharma, China) were transfected using the Lipofectamine™ RNAi MAX protocol from GenePharma. Small interfering RNA (siRNA) target sequences for FBXO1 were as follows: si‐FBXO1#1, sense: 5′-GCUCUUUCACAUCCUGAAATT-3′; si‐FBXO1#2, 5′- GCUGCAGAGGACUCACAAATT-3′; Negative Control (NC), sense: 5′-UUCUCCGAACGUGUCACGUTT-3′. Western blotting, RT-qPCR and fluorescence modification were used to determine the efficiency of siRNA knockdown.

### RNA extraction and Real-time quantitative PCR

Total RNA extracted from MCF-7 and MDA-MB-231 cells with Trizol Reagent (Invitrogen, Carlsbad, CA, USA) were reverse transcribed with RT reagent Kit gDNA Eraser (TaKaRa). Next, SYBR-Green (TaKaRa) and qRT-PCR analysis were used for detecting cDNA expression levels and β-ACTIN was used as internal reference. Primers were shown as follows: β-ACTIN, Forward (F): 5′-TGGCACCCAGCACAATGAA-3′, Reverse(R): 5′-CTAAGTCATAGTCCGCCTAGAAGCA-3′; hFBXO1, Forward (F): 5′-ATGGCTCACGGACAACACTT-3′, Reverse (R): 5′-TGGGGACTCGAATCTTCCCT-3′.

### Western blotting

Total proteins were extracted using radioimmunoprecipitation buffer (pH 7.4, 150 mM NaCl, 25 mM Tris, 1% Nonidet P‐40, 0.5% sodium deoxycholate, 0.1% sodium dodecyl sulfate [SDS]) supplemented with protease inhibitor (Roche, Basel, Switzerland). Quantitative analysis of protein content was measured by the BCA kit (Tiangen, China) and separated using 10% sodium dodecyl sulfate‐polyacrylamide gel electrophoresis. The separated proteins were transferred to nitrocellulose membranes and blocked in 5% nonfat milk. The membranes were incubated with primary antibodies overnight at 4 °C, including FBXO1, α, β-Tubulin (Cell Signaling Technology, Danvers, MA, USA) and Vinculin (Abcam, Cambridge, MA, USA). After washing, the membranes were incubated with fluorophore‐conjugated secondary antibodies. Odyssey Scanner (Li‐Cor, Bioscience, Lincoln, NE) was used to visualize the blots.

### Cell viability and colony formation assay

Cell viability was detected by CCK-8 assay using a kit provided by Dojindo Molecular Technologies. Cell suspension with a cell density of 4 × 10^3^ cells/ml was prepared using cells of two cell lines. Then 4 × 10^3^ cells in 0.1 ml cell suspension were used to fill each well of a 96-well plate. Cells were cultured at 37 ºC with 5% CO_2_, and 10 μl of CCK-8 was added into each well at 24, 48 and 72 h later. Cells were cultured for another 4 h and a microplate reader was used to measure OD values at 450 nm. In colony formation assay, cells (10^4^ cells/well) were seeded in 6-well plate and supported for 7–14 days in a humidified incubator with 37 °C, 5% CO_2_ until colonies of cells appeared. The colonies were fixed with methanol and stained with 0.5% crystal violet in order to be counted.

### Transwell migration and wound‐healing assay

The migration assay was accessed by transwell chamber with 8 μm pores (Corning Incorporated, NY, USA). Breast cancer cells seeded in six‐well plates were cultivated with negative control and si‐FBXO1 for 48 h. After the transfection experiment, 5 × 10^3^ cells were seeded on the Matrigel in 100 μl of medium with 0.1% fetal bovine serum (FBS). The lower chamber was added 400 μl medium with 10% FBS. Invasive cells were then stained with 0.5% crystal violet and observed. Wound‐healing assay was used to assess the ability of cell migration, and wounds were made by 200 μl pipette tip. Images were taken at 0, 24, and 48 h with the microscope (Leica, DMI1). Migration distance was analyzed using the ImageJ software (National Institutes of Health, Bethesda, MD).

### Statistical analysis

Two‐tailed Student t test and analysis of variance were performed, respectively, to compare the differences between the data of two groups. Each experiment was repeated three times or more and all data were presented as mean ± standard deviation (SD). All statistical analyses were performed using the SPSS version 24.0 (SPSS Inc, Chicago, IL) and GraphPad Prism 8.0 software package (GraphPad Software Inc, SanDiego, CA). Statistical significance was described as follows: *P < 0.05; **P < 0.01; ***P < 0.001; ****P < 0.0001.

## Results

### Significant transcriptional levels of FBXOs in BC

**Fig. 1:**
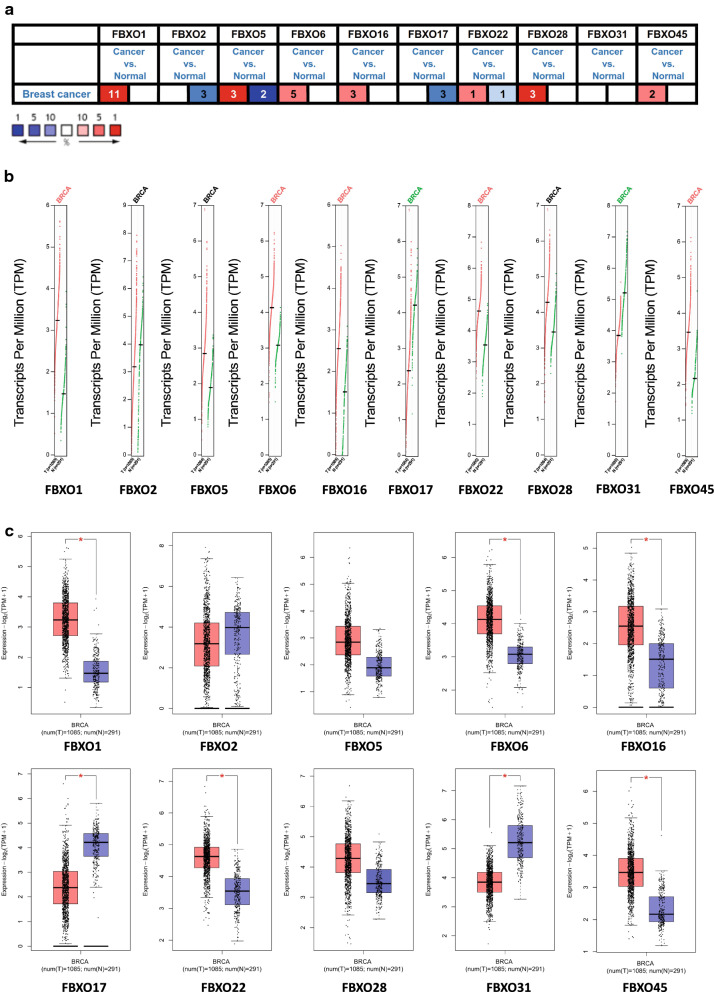
The transcription levels of 10 FBXO members in Breast Cancer. **a** The Expression of FBXOs in BC in Oncomine Database. Red, over-expression; Blue, down-regulated expression. **b** The scatter diagram of Expression of FBXOs in BC in GEPIA Database. **c** The box plot of Expression of FBXOs in BC in GEPIA Database. T, Tumor tissues; N, Normal tissues; * P-value was set at 0.01

In order to explore the prognostic and potential therapeutic values of different FBXO members in BC, the ONCOMINE databases were used to compare the mRNA expression levels of FBXOs in BC samples with normal breast samples (Fig. 1). Ten FBXO genes were identified within the human BC cells. According to our findings, FBXO1, 2, 5, 6, 16, 17, 22, 28 and 45 were remarkably altered in different types of BC cells. FBXO1, 6, 16, 28, and 45 were all expressed at high levels in various pathological types of BC. FBXO2 and 17 were significantly downregulated in different types. As for FBXO5 and 22, they showed the contrary expression pattern. The specific fold change, p-value, and the value of t-test of different significantly statistical analysis were showed in Table 1 [8, 22, 32–36]. Using ONCOMINE and UALCAN databases, we compared the expression situations of FBXOs in more than 20 types of tumor and normal samples across TCGA datasets to explore the FBXOs’ regular pattern of expression (Additional file 1: Figure S1, Additional file 2: Figure S2).

**Table 1:** The significant changes of FBXOs transcription levels between different types of BC and normal breast tissues (oncomine)

		Type of breast cancer versus normal breast tissue	Fold change	p value	t Test	Source and/or reference
FBXO1		Medullary Breast Carcinoma	2.693	2.57E−14	11.576	Curtis Breast Statistics [32]
		Invasive Ductal Breast Carcinoma	2.262	2.21E−71	28.235	Curtis Breast Statistics[32]
		Invasive Breast Carcinoma	2.220	9.12E−22	11.387	TCGA Breast Statistics
		Invasive Ductal Breast Carcinoma	2.704	5.66E−33	19.032	TCGA Breast Statistics
		Intraductal Cribriform Breast Adenocarcinoma	3.188	6.24E−06	11.863	TCGA Breast Statistics
		Mixed Lobular and Ductal Breast Carcinoma	2.093	2.75E−05	6.958	TCGA Breast Statistics
		Invasive Lobular Breast Carcinoma	2.091	1.66E−11	7.626	TCGA Breast Statistics
		Male Breast Carcinoma	2.666	8.42E−04	9.609	TCGA Breast Statistics
		Mucinous Breast Carcinoma	3.057	5.00E−03	5.182	TCGA Breast Statistics
		Invasive Ductal Breast Carcinoma	3.014	8.00E−03	2.923	Radvanyi Breast Statistics[33]
		Invasive Mixed Breast Carcinoma	3.200	8.00E−03	2.886	Radvanyi Breast Statistics[33]
FBXO2		Invasive Lobular Breast Carcinoma	− 3.055	6.00E−03	− 3.526	Turashvili Breast Statistics[22]
		Invasive Ductal Breast Carcinoma	− 2.341	3.00E−03	− 3.693	Turashvili Breast Statistics[22]
		Ductal Breast Carcinoma	− 4.181	5.28E−07	− 6.227	Richardson Breast 2 Statistics[34]
FBXO5		Medullary Breast Carcinoma	2.727	1.30E−17	15.376	Curtis Breast Statistics[32]
		Ductal Breast Carcinoma	2.844	9.10E−10	8.321	Richardson Breast 2 Statistics[34]
		Invasive Ductal Breast Carcinoma	2.210	2.00E−03	3.638	Radvanyi Breast Statistics[33]
		Invasive Lobular Breast Carcinoma	− 2.746	3.00E−03	− 3.196	Turashvili Breast Statistics[22]
		Invasive Breast Carcinoma Stroma	− 5.547	8.32E−17	− 14.103	Finak Breast Statistics[35]
FBXO6		Medullary Breast Carcinoma	2.547	1.47E−12	10.480	Curtis Breast Statistics[32]
		Mixed Lobular and Ductal Breast Carcinoma	2.050	2.29E−05	7.560	TCGA Breast Statistics
		Intraductal Cribriform Breast Adenocarcinoma	2.271	8.85E−05	12.075	TCGA Breast Statistics
		Invasive Breast Carcinoma	2.106	1.51E−18	10.139	TCGA Breast Statistics
		Ductal Breast Carcinoma	2.874	3.19E−05	5.031	Richardson Breast 2 Statistics[34]
FBXO16		Invasive Breast Carcinoma Stroma	3.077	2.43E−15	14.718	Finak Breast Statistics[35]
		Invasive Ductal and Lobular Carcinoma	4.129	7.68E−04	7.210	TCGA Breast Statistics
		Mixed Lobular and Ductal Breast Carcinoma	2.969	8.15E−04	4.384	TCGA Breast Statistics
FBXO17		Invasive Ductal Breast Carcinoma Stroma	− 2.266	6.53E−04	− 3.755	Karnoub Breast Statistics[8]
		Mixed Lobular and Ductal Breast Carcinoma	− 2.472	1.63E−04	− 5.846	TCGA Breast Statistics
		Intraductal Cribriform Breast Adenocarcinoma	− 2.787	6.00E−03	− 5.243	TCGA Breast Statistics
FBXO22		Invasive Ductal Breast Carcinoma Stroma	6.235	2.03E−04	4.281	Karnoub Breast Statistics[8]
		Invasive Breast Carcinoma Stroma	− 2.451	3.33E−19	− 16.952	Finak Breast Statistics[35]
FBXO28		Invasive Ductal Breast Carcinoma	2.274	1.00E−03	3.412	Turashvili Breast Statistics[22]
		Ductal Breast Carcinoma in Situ Epithelia	2.349	6.14E−04	4.100	Ma Breast 4 Statistics[36]
		Ductal Breast Carcinoma	2.628	5.05E−06	7.146	Richardson Breast 2 Statistics[34]
FBXO31		NA	NA	NA	NA	NA
FBXO45		Ductal Breast Carcinoma	2.705	1.27E−07	7.433	Richardson Breast 2 Statistics[34]
		Ductal Breast Carcinoma in Situ Epithelia	2.654	5.00E−03	3.259	Ma Breast 4 Statistics[36]

*NA* not available, *TCGA* The Cancer Genome Atlas

Next, we explored the distinction between the mRNA expression of FBXO family members and normal breast tissues in different subcategory of breast invasive carcinoma (BRCA) in GEPIA database. The overall results indicated that the expression levels of FBXO1, FBXO6, FBXO16, FBXO22 and FBXO45 in BRCA were higher than those in normal tissues, and the expression levels of FBXO17 and FBXO31 were lower in BRCA samples (Fig. 1).

### The correlation between mRNA expression levels of FBXOs and clinicopathological parameters of BC

**Fig. 2:**
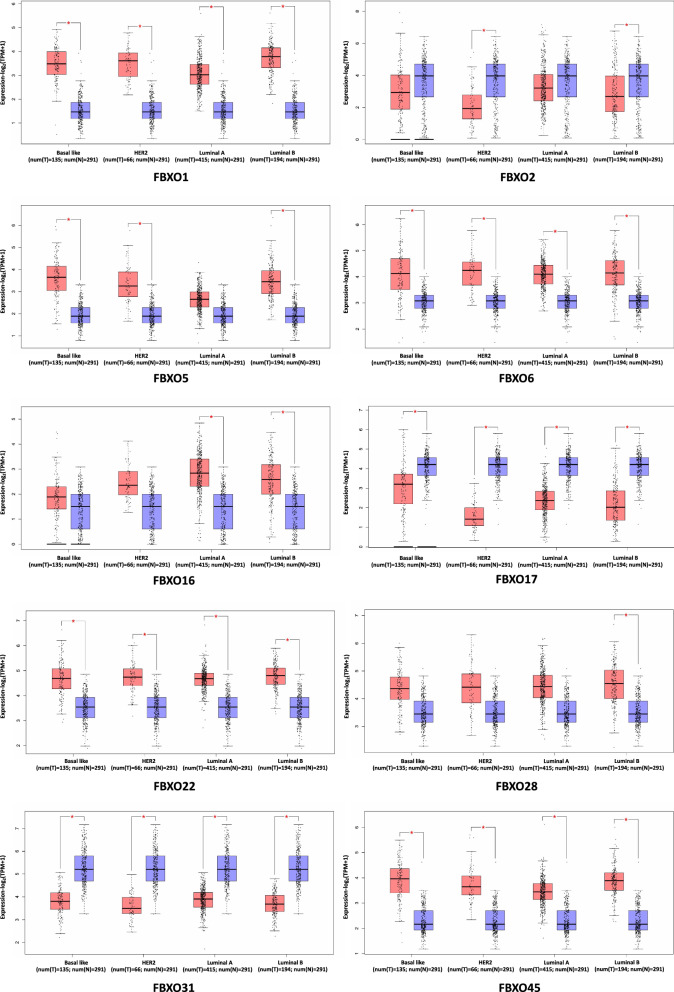
The Transcriptional Levels of FBXOs in Different Subtypes of BC in GEPIA Database. T, Tumor tissues; N, Normal tissues; * P-value was set at 0.01

We analyzed the transcriptional levels of FBXOs in different molecular subtypes of BRCA by using GEPIA database, and all data was from TCGA and GTEx datasets. Significantly increased FBXO1, FBXO6, FBXO22, and FBXO45 were observed in all BRCA subtypes compared with normal breast groups. The expression levels of FBXO17 and FBXO31 were significantly decreased in all BRCA subtypes. As for FBXO2, it was found expressed lower in HER2 and luminal B subtypes of BRCA. The mRNA of FBXO5 showed up-regulated in Basal-like, HER2 and luminal B subtypes. In luminal-types breast carcinoma, FBXO16 was inclined to over-express in luminal A and B groups and FBXO28 was a potential up-regulated biomarker of luminal B groups (Fig. 2).

**Fig. 3:**
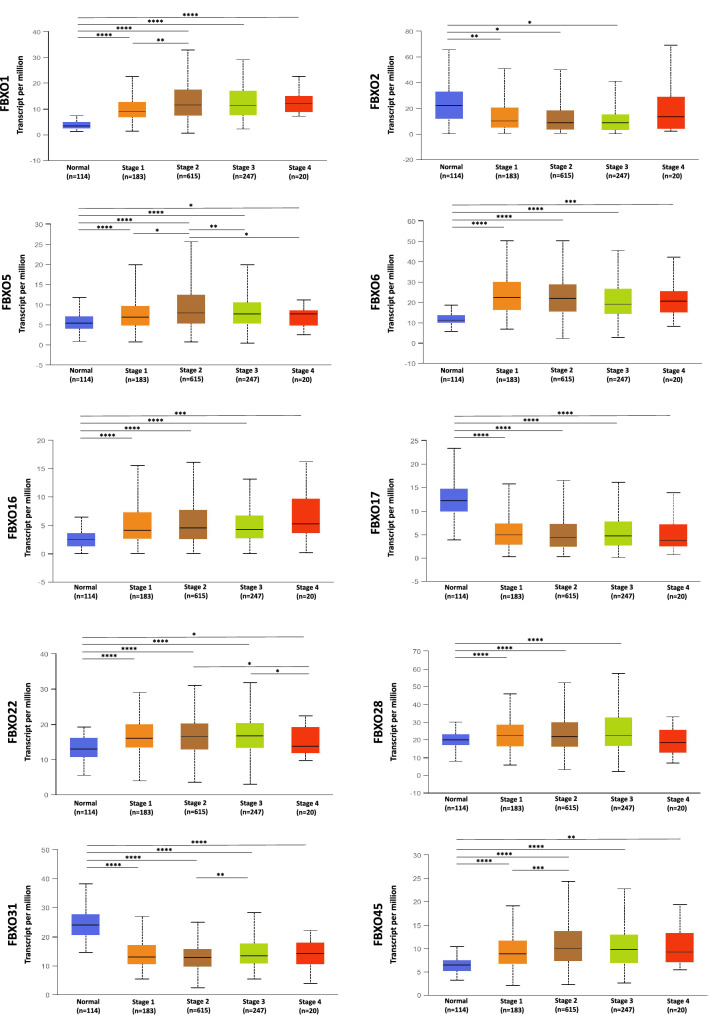
Correlation between FBXOs Expression and Tumor Stage of BC Patients in UALCAN Database. *P < 0.05; **P < 0.01; *** P < 0.001; ****P < 0.0001

Based on aforesaid research, we probed into the correlation between the mRNA expression of FBXOs and clinicopathological stage of BRCA patients via UALCAN database. In all family members, there were considerable differences of transcriptional levels between normal groups and the patient groups divided by different pathological stages. Among the results, FBXO1, FBXO5, FBXO6, FBXO16, FBXO22, FBXO28 and FBXO45 were up-regulated in the pathological stage groups, FBXO2, FBXO17 and FBXO31 were negative expression factors in BRCA patients. More details of expression differences were showed in Fig. 3, P < 0.05 was considered to be statistically significant (*P < 0.05; **P < 0.01; ***P < 0.001; ****P < 0.0001).

We also used bc-GenExMiner (v4.4) online tool to assess the relationship between FBXOs expression levels and various clinical features of BC patients based on RNA-seq technology (Table 2). The clinical features include age, nodal metastasis status, ER/PR/HER2 status, basal-like statues, triple-negative statues (TNBC), P53 status, Scarff Bloom & Richardson grade status (SBR) and Nottingham Prognostic Index (NPI). The table showed clearly that both of FBXO1 and FBXO45 had significant high-expression differences in the clinical patient groups of younger age (Age < 51), lymph nodes metastasis, ER (−), PR (−), HER2 ( +), basal-like subtype, triple-negative subtype, P53 gene mutation, level III of SBR and level III of NPI. The results implied that FBXO1 and FBXO45 were positively correlated with the types of highly malignant and poor-prognostic BC, which have the features of low differentiation, high invasiveness, easy to metastasize and relapse. It means that FBXO1 and FBXO45 could be potential biomarkers to identify special types of BC.

**Table 2:** Associations of FBXOs expression with clinical manifestations in BC patients (Bc-GenExMiner, v4.4)

Variables	**No**	**FBXO1**	*P-value*	**FBXO2**	*P-value*	**FBXO5**	*P-value*	**FBXO6**	*P-value*	**FBXO16**	*P-value*	**FBXO17**	*P-value*	**FBXO22**	*P-value*	**FBXO28**	*P-value*	**FBXO31**	*P-value*	**FBXO45**	*P-value*
Age																					
≤ 51	1099	↑	****	**–**	p = .0515	↑	****	**–**	p = .7262	**–**	p = .7978	↑	***	**–**	p = .6565	**–**	p = .6215	↑	**	↑	****
> 51	3208			**–**				**–**		**–**				**–**		**–**					
Nodal status																					
–	2415		***	**–**	p = .6545	**–**	p = .2297	**–**	p = .1867	**–**	p = .2105	**–**	p = .3476		****		*	**–**	p = .2323		*
+	1646	↑		**–**		**–**		**–**		**–**		**–**		↑		↑		**–**		↑	
ER(IHC)																					
–	551	↑	****		****	↑	****	↑	**		****	↑	****	↑	*		***	**–**	p = .2564	↑	****
+	3911			↑						↑						↑		**–**			
PR(IHC)																					
–	828	↑	****		****	↑	****	**–**	p = .2212		****	↑	*	**–**	p = .1015	**–**	p = .4141	**–**	p = .4587	↑	****
+	3498			↑				**–**		↑				**–**		**–**		**–**			
HER2(IHC)																					
–	3582		****	↑	****		****	**–**	p = .4834	↑	****	↑	****	**–**	p = .5320	**–**	p = .2814	↑	****		****
+	661	↑				↑		**–**						**–**		**–**				↑	
Basal-like statues																					
Basal-like	832	↑	****		**	↑	****	↑	***		****	↑	****	**–**	p = .2002		***	↑	*	↑	****
Not	3836			↑						↑				**–**		↑					
Triple-negative statues(IHC)																					
TNBC	317	↑	****		****	↑	****	**–**	p = .4260		****	↑	****	↑	*		**	**–**	p = .7470	↑	****
Not	4119			↑				**–**		↑						↑		**–**			
P53 status (sequence-based)																					
Wild type	699		****	↑	***		****	**–**	p = .2017	↑	****		***	**–**	p = .7978	**–**	p = .6453	**–**	p = .0796		****
Mutated	328	↑				↑		**–**				↑		**–**		**–**		**–**		↑	
SBR																					
SBR1	544	SBR2 > SBR1	****	SBR3 < SBR1	****	SBR3 > SBR1	****	SBR3 > SBR1	****	SBR2 < SBR1	****	SBR2 < SBR1	****	SBR3 > SBR1	****	**–**	p = .0730	SBR3 < SBR1	****	SBR2 > SBR1	****
SBR2	1699	SBR3 > SBR1	****	SBR3 < SBR2	****	SBR3 > SBR2	****	SBR3 > SBR2	****	SBR3 < SBR1	****	SBR3 < SBR1	****	SBR2 > SBR1	*	**–**		SBR3 < SBR2	****	SBR3 > SBR1	****
SBR3	1374	SBR3 > SBR2	****	SBR2 = SBR1	**–**	SBR2 > SBR1	****	SBR2 > SBR1	**	SBR3 < SBR2	****	SBR3 < SBR2	**	SBR3 > SBR2	*	**–**		SBR2 < SBR1	***	SBR3 > SBR2	****
TPI																					
NPI1	1173	NPI2 > NPI1	****	NPI2 < NPI1	*	NPI2 > NPI1	****	NPI2 > NPI1	****	NPI2 < NPI1	****	NPI2 < NPI1	****	NPI3 > NPI1	****	NPI3 > NPI1	*	NPI2 < NPI1	****	NPI2 > NPI1	****
NPI2	1525	NPI3 > NPI1	****	NPI3 < NPI1	*	NPI3 > NPI1	****	NPI3 > NPI1	****	NPI3 < NPI1	****	NPI3 < NPI1	****	NPI3 > NPI2	**	NPI2 = NPI1	**–**	NPI3 < NPI1	****	NPI3 > NPI1	****
NPI3	416	NPI3 > NPI2	****	NPI3 = NPI2	**–**	NPI3 > NPI2	**	NPI3 > NPI2	*	NPI3 = NPI2	**–**	NPI3 = NPI2	**–**	NPI2 > NPI1	*	NPI3 = NPI2	**–**	NPI3 < NPI2	**	NPI3 > NPI2	***

↑, upregulated; –, no significant difference; No., number of patients enrolled; ER, oestrogen receptor; PR, progesterone receptor; HER2, human epidermal growth factor receptor 2; TNBC, triple-negative breast cancer; IHC, immunohistochemistry; SBR, Scarff-Bloom-Richardson grade; NPI, Nottingham prognostic index

^*^P < 0.05; **P < 0.01; *** P < 0.001; ****P < 0.0001

**Fig. 4:**
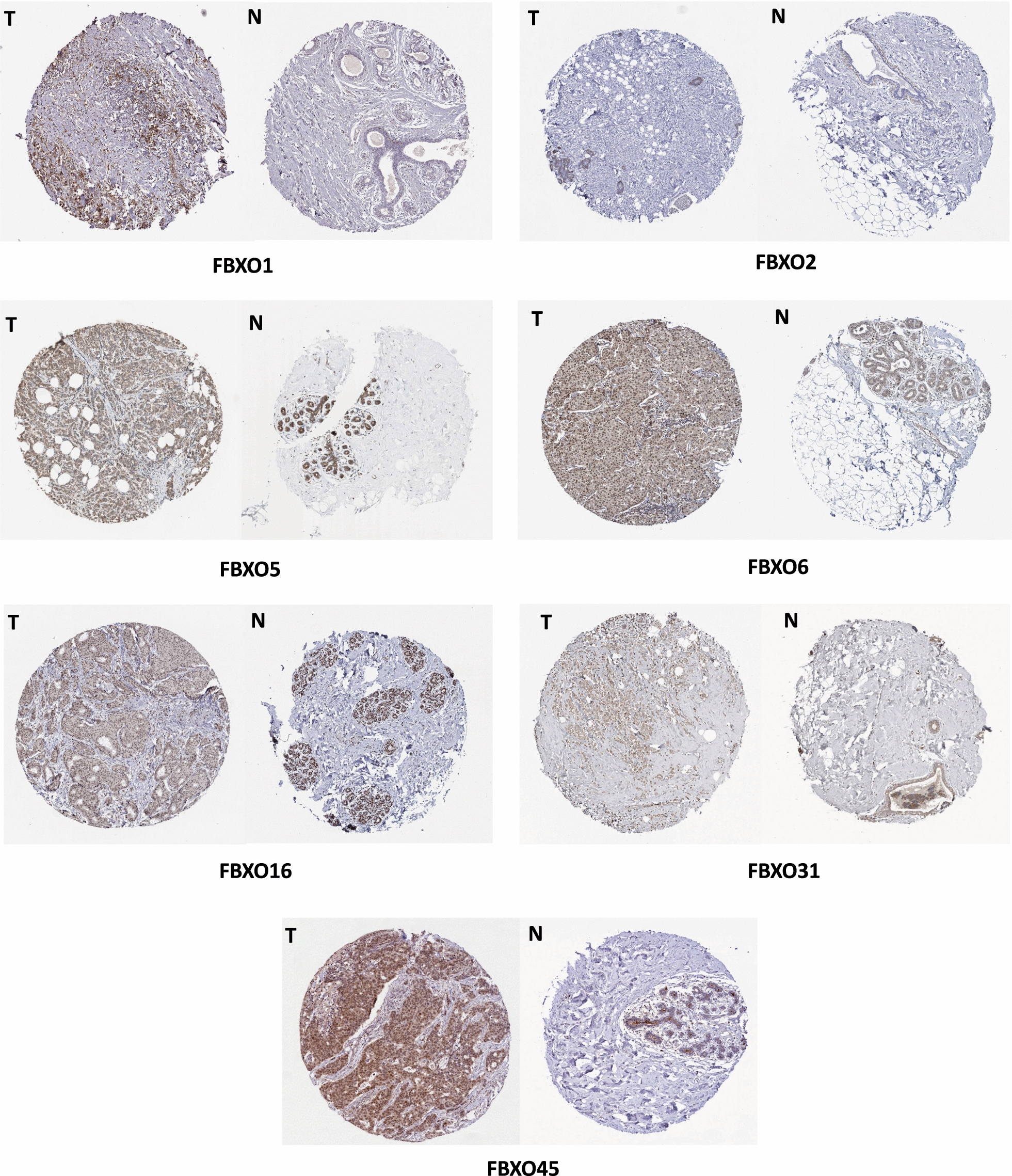
The Comparison of Protein Expression of FBXOs between BC and Normal Tissues from Human Protein Atlas (HPA). FBXO1, FBXO5, FBXO6, FBXO16, FBXO45 proteins were highly expressed in BC than in the normal tissues. The expression differences of FBXO2 and FBXO31 were not obvious between tumor and normal tissues

Next, we provided the immunohistochemistry (IHC) outcomes from HPA database to verify the difference of protein expression of FBXO family members from HPA database. We found that FBXO1, FBXO5, FBXO6, FBXO16, FBXO45 proteins were more highly expressed in the BC tissues than those in the normal tissues. The expression differences were not obvious of FBXO2 and FBXO31. The IHC results of FBXO17, FBXO22, FBXO28 need to be further updated in HPA database (Fig. 4).

### The genetic alteration and mutation information of FBXO family members

**Fig. 5:**
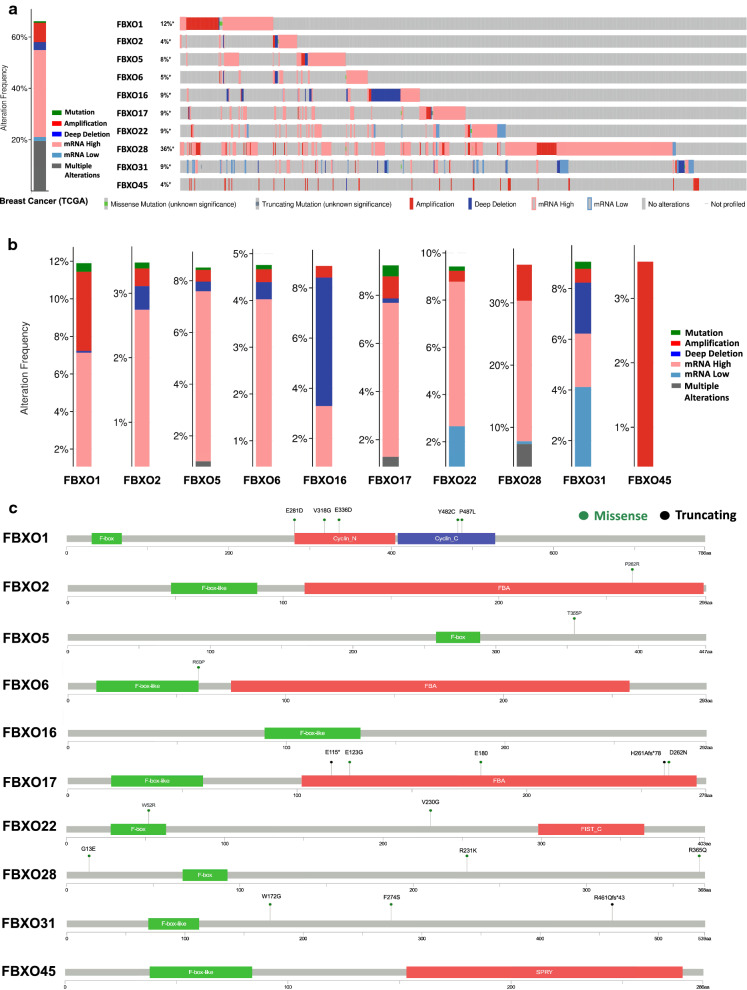
The mutation information of FBXO genes in human BC (cBioPortal). **a** Alteration frequency of 10 FBXO genes at the overall and individual levels. **b** The bar graph of gene alteration frequency of FBXO genes. **c** Schematic representation of gene mutation sites of FBXOs on the coding strand

We analyzed the FBXO genes’ alterations and mutation situation in the cBioPortal online tool for breast invasive carcinoma (TCGA, Firehose Legacy). As showed in Fig. 5a, target genes were altered in 723 of 1093 patient cases with the percent of 66.15%. The highest frequency of alterations was found in FBXO28 (395 of 1093 samples, 36.14%), with mRNA up-regulation of 22.6% (247 cases), genetic amplification of 5.76% (63 cases), mRNA down-regulation of 0.46% (5 cases) and other multiple alterations of 7.32% (80 cases) (Fig. 5a, b). The second gene was FBXO1, and it altered in 11.89% of 1093 patient cases. The main genetic alterations involved mRNA up-regulation (70 cases, 6.4%), genetic amplification (46 cases, 4.21%), mutation (5 cases, 0.46%), deep deletion (1 case, 0.09%) and other multiple alterations (8 cases, 0.73%) (Fig. 5a, b). Other gene alterations included FBXO2 (38 of 1093 samples, 3.48%), FBXO5 (93 of 1093 samples, 8.51%), FBXO6 (52 of 1093 samples, 4.76%), FBXO16 (95 of 1093 samples, 8.87%), FBXO17 (101 of 1093 samples, 9.24%), FBXO22 (103 of 1093 samples, 9.42%), FBXO31 (99 of 1093 samples, 9.06%) and FBXO45 (39 of 1093 samples, 3.57%). The specific percentage of each gene alteration is shown in Fig. 5b. The largest proportion of alterations was high mRNA expression, especially in FBXO2, FBXO5, FBXO6, FBXO17 and FBXO22. Interestingly, there was no overexpression of mRNA was detected in FBXO45, but it had high frequency of genetic amplification of 3.48% (38 cases). Furthermore, we extracted the gene mutation information of the FBXOs from cBioPortal website tool. The overall somatic mutation frequency was very low. The frequency of FBXO1 and FBXO17 was 0.5%, the frequency of FBXO28 and FBXO31 was 0.3%, the rest members’ mutation frequency was no more than 0.2%. Figure 5c displayed the specific mutation site in FBXOs DNA sequences. The green dots indicate missense mutations and the black ones mean truncating sites. These results illustrated that the ten FBXOs members had excellent genetic stability as potential BC universal biomarkers.

### Prognostic values of FBXOs’ mRNA expression levels in BC patients

**Fig. 6:**
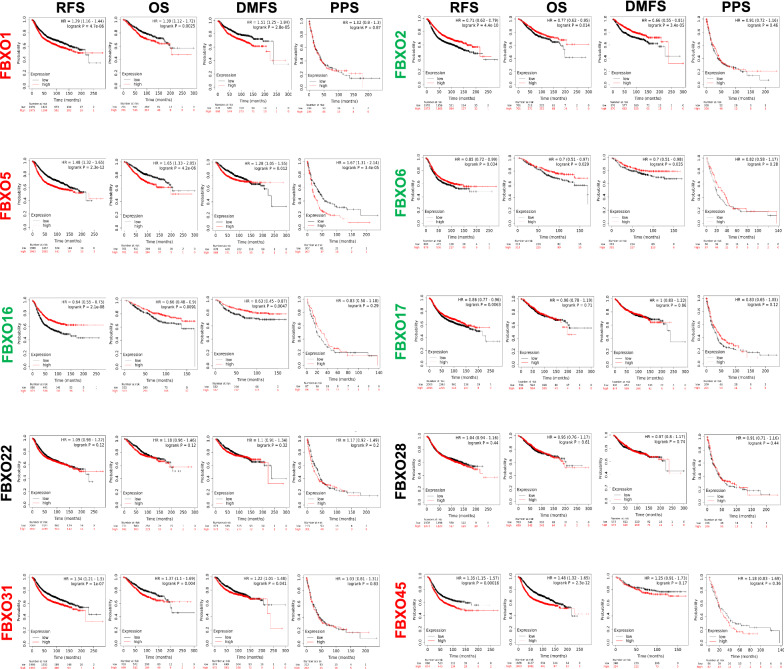
The prognostic values of FXBO family members in BC patients. The survival curves comparing BC patients with high (red) and low (black) FBXO expression levels were plotted using the Kaplan–Meier Plotter. DFS, disease-free survival rate; OS, the overall survival rate; DMFS, distance metastasis free survival; PPS, post progression survival; The threshold P-value is less than 0.05

In order to evaluate the clinical significance of FBXOs, we used publicly Kaplan–Meier Plotter tools to explore the correlation between FBXO family members’ transcriptional level and the survival of patients with overall BC and different molecular subtypes of BC patients further. The main parameters of survival analysis include relapse free survival (RFS), overall survival (OS), distant metastasis free survival (DMFS) and post progression survival (PPS). Survival curves according to Kaplan‐Meier showed in Fig. 6, suggesting that high mRNA levels of FBXO1, 5, 31 and 45 were significantly associated with worse prognosis in BC patients. By contrast, high transcription levels of FBXO2, 6, 16, 17 symbolized a better prognosis of BC patients (P < 0.05). Moreover, we found that increased expression of FBXO1 mRNA revealed a significant correlation with worse RFS, OS and DMFS in overall BC patients (Fig. 6), as well as in luminal A subtype (Additional file 3: Figure S3). The high mRNA levels of FBXO2 was significantly associated with better RFS, OS and DMFS in overall BC patients (Fig. 6). In luminal B and HER2 subtypes, FBXO2 symbolized a better prognosis similarly (Additional file 3: Figure S3). The increased transcriptional levels of FBXO5 were related to poor RFS, OS, DMFS and PPS in overall BC and luminal A subtype patients (Fig. 6). In luminal B and triple-negative subtypes, high expression of FBXO5 was related to poor RFS (Additional file 3: Figure S3). FBXO6 is a marker for good prognosis of BC patients, high mRNA level of FBXO6 meaning better RFS, OS, DMFS in overall patient groups (Fig. 6). Increased expression of FBXO6 was related to better RFS in HER2 subtypes and better RFS, OS in TNBC (Additional file 3: Figure S3). As for FBXO16 and FBXO17, they are favorable prognosis markers in BC (Fig. 6). High transcriptional levels of FBXO16 was associated with better RFS and OS in luminal A and better RFS in luminal B of BC groups (Additional file 3: Figure S3). Increased mRNA levels of FBXO17 revealed a significant correlation with better PPS in HER2 BC patients (Additional file 3: Figure S3). The overexpression of FBXO22 was only related to worse OS in HER2 BC (Additional file 3: Figure S3) and overexpression of FBXO28 was only related to worse RFS in luminal B and TNBC types of BC (Additional file 3: Figure S3). High transcription level of FBXO31 was interrelated with poor RFS and OS in overall BC and poor RFS is luminal A, luminal B and HER2 subtypes patients (Additional file 3: Figure S3). FBXO45 is a poor prognosis marker in BC. We found that it was related to worse RFS and OS in overall BC when overexpressed. In luminal subtypes of BC, high transcriptional level of it also suggested poor RFS and OS (Additional file 3: Figure S3). In a conclusion, FBXO2, FBXO6, FBXO16 and FBXO17 were potential favorable prognostic factors for BC. FBXO1, FBXO5, FBXO22, FBXO28, FBXO31 and FBXO45 may be the independent poor prognostic factors in BC.

### Functional enrichment analysis of FBXO1 and co-expressed genes in BC

**Fig. 7:**
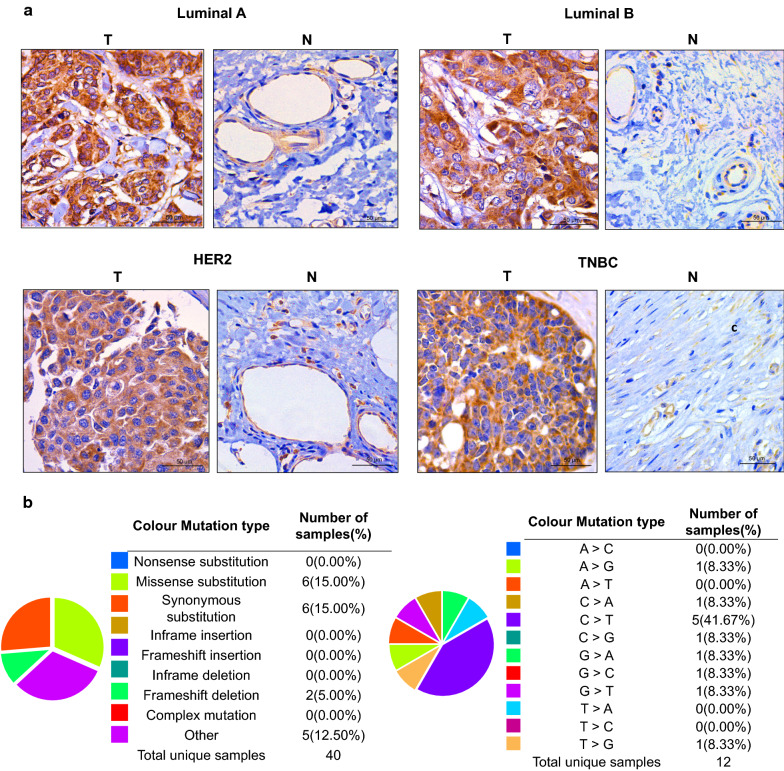

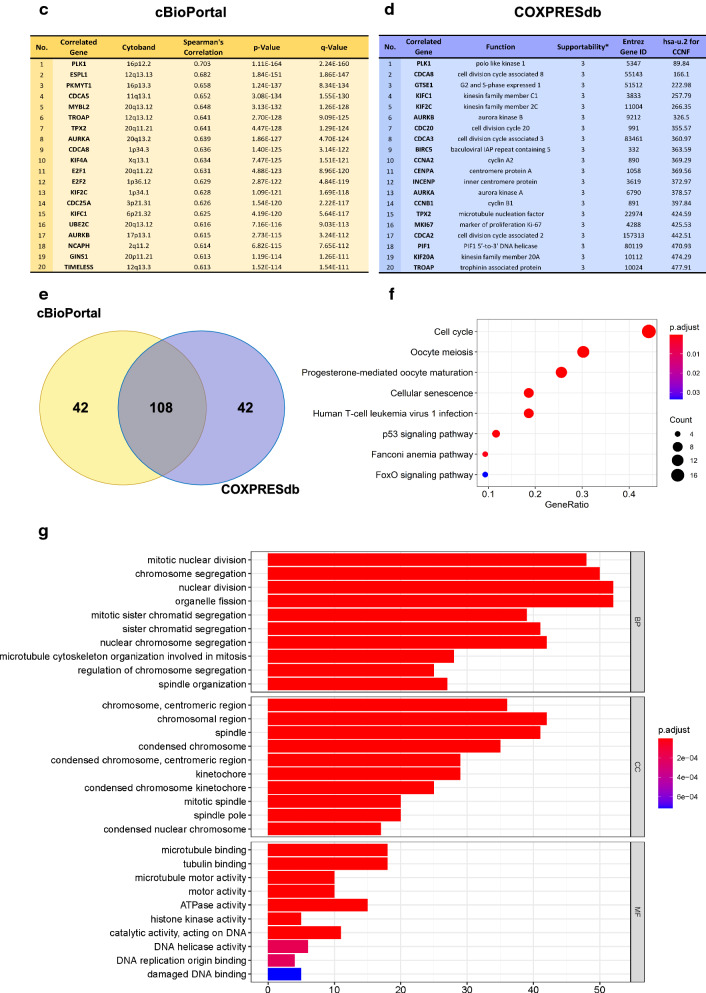
Comprehensive Bioinformatics Analysis of FBXO1 and Co-expressed Genes in BC. **a** High expression of FBXO1 protein in different subtypes of BC verified by immunohistochemistry. Scale bar = 50 μm, T, tumor tissues; N, normal tissues. **b** The percentages of mutation types of FBXO1 in BC were indicated in a pie chart generated from Catalogue of Somatic Mutations in COSMIC database. **c**, **d** The top 150 genes positively associated with FBXO1 transcript level based on the cBioPortal and COXPRESdb databases in BC. The tables listed the top 20 genes. **e** Venn diagram represented the intersection of top positively corrected genes between the cBioPortal and COXPRESdb databases. **f** The bubble diagram showed the functions of FBXO1 and 108 genes significantly associated with FBXO1 alterations, which were predicted by the analysis of Kyoto Encyclopedia of Genes and Genomes (KEGG) by STRING tools. **g** Gene Ontology (GO) enrichment analysis predicted the functional roles of FBXO1 and 108 co-expressed genes based on three aspects, including biological processes (BP), cellular components (CC) and molecular function (MF)

All our preliminary results throw light on the importance of FBXO1. As a novel biomarker in human BC, FBXO1 may play a crucial role in the process of tumorigenesis and development and may be the potential target of precision therapy for patients with BC. We further performed the IHC staining in clinical different molecular subtypes of BC tissues to verify the expression situation of FBXO1 protein. Our IHC results showed that significantly increased FBXO1 was observed highly expressed in all clinical subtypes of BC tissues than in the normal tissues (Fig. 7a). The additional clinical information of samples used in IHC assay was showed in Additional file 4: Table S1. Next, we analyzed the specific mutations of FBXO1 in BC by employing the COSMIC database. The largest proportion of mutations were missense substitution (15%) and synonymous substitution (15%). The largest proportion of nucleotide changes was C > T (41.67%), the rest included 8.33% of A > G, C > A, C > G, G > A, G > C, G > T and T > C (Fig. 7b). Then We screened the top 150 co-expressed genes that were most related to FBXO1 from the cBioPortal and COXPRESdb online tools. The top 20 genes from both databases were displayed in Fig. 7c, d. We obtained a cohort of 108 crossed genes shown by Venn diagram in Fig. 7e.

GO enrichment analysis indicated that the biological processes (BP) including mitotic nuclear division, chromosome segregation, nuclear division and organelle fission were mostly significantly regulated by the FBXO1 and co-expressed genes alterations in breast adenocarcinoma. Mostly significant cell component (CC) included chromosomal region, spindle, centromeric region and condensed chromosome. Besides, as molecular function (MF), microtubule binding, tubulin binding and ATPase activity were mostly significantly affected by targeted genes in Fig. 7g. KEGG analysis demonstrated the pathways were mostly correlated with the functions of FBXO1 and co-expressed genes shown in bubble chart (Fig. 7f). Cell cycle (hsa04110) was considered as the most relevant pathway which FBXO1 and co-expressed genes participated in Table 3. Furthermore, using DAVID database, we marked the key points regulated by FBXO1 and co-expressed genes alteration refer to Additional file 5: Figure S4. Collectively, through experiment and database analysis, FBXO1 protein was truly increased in BC tissues. It may be an excellent therapeutic target for clinical BC patients because the stability of FBXO1 gene is of a high degree and the mutations are very rare. Moreover, GO and KEGG analysis suggested that FBXO1 and 108 co-expressed genes may play essential roles in regulating the tumorigenesis and proliferation in BC.

**Table 3:** KEGG Enrichment Analysis of Co-expressed Genes with FBXO1

ID	Description	Count	p-value	p.adjust
hsa04110	Cell cycle	19	3.54E−24	1.27E−22
hsa04114	Oocyte meiosis	13	4.56E−14	8.20E−13
hsa04914	Progesterone-mediated oocyte maturation	11	2.00E−12	2.40E−11
hsa04218	Cellular senescence	8	1.52E−06	1.37E−05
hsa05166	Human T-cell leukemia virus 1 infection	8	1.57E−05	1.13E−04
hsa04115	p53 signaling pathway	5	3.78E−05	2.27E−04
hsa03460	Fanconi anemia pathway	4	1.78E−04	9.17E−04
hsa04068	FoxO signaling pathway	4	4.92E−03	2.22E−02

### Knockdown of FBXO1 suppresses the proliferation and migration of breast cancer cells

**Fig. 8:**
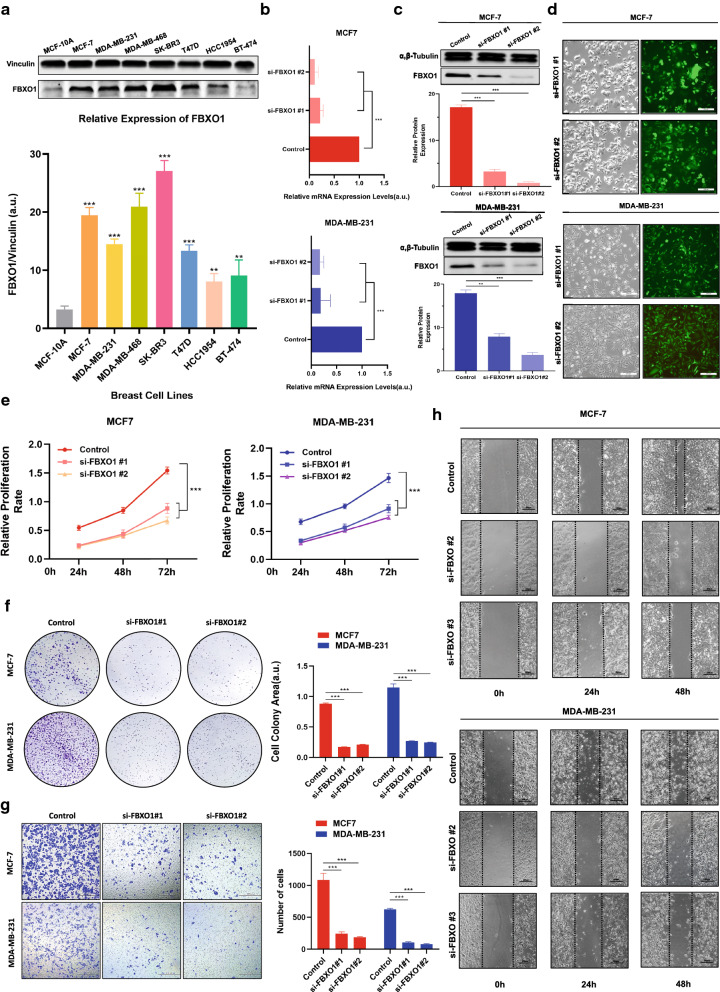
The knockdown of FBXO1 attenuates the proliferation and migration of breast cancer cells in vitro. **a** Upper panel, the expression levels of FBXO1 protein examined by Western blotting in 8 human breast cell lines. Lower panel, bar graphs representing quantification of Western blotting bands. **b** Determination of relative mRNA expression levels of FBXO1 in control and si‐FBXO1‐transfected MCF7 and MDA-MB-231 breast cancer cell lines by RT-qPCR assay. **c** Immunoblotting analyses of proteins as indicated in control and si‐FBXO1‐transfected MCF7 and MDA-MB-231 cell lines**,** bar graphs representing quantification of Western blotting bands. **d** Diagram of successful transfection of siRNA of FBXO1 labeled by FAM fluorescence dye in MCF7 and MDA-MB-231 cell lines. **e** The knockdown of FBXO1 attenuates the proliferation of breast cancer cells in vitro. Cell Counting Kit-8 assay showed the relative proliferative capacity of specific MCF7 and MDA-MB-231 cells at 24, 48, and 72 h after seeding in plates. **f** The knockdown of FBXO1 attenuates the proliferation of breast cancer cells in vitro. Colony-forming assay showed the relative proliferative capacity of specific MCF7 and MDA-MB-231 cells at 48 h after seeding in plates(left) and quantification of the colony areas (right). **g** The knockdown of FBXO1 attenuates the migration of breast cancer cells in vitro. Transwell migration assay showed representative images of specific MCF7 and MDA-MB-231 cells (left) and quantification of the cell numbers (right). **h** The knockdown of FBXO1 attenuates the migration of breast cancer cells in vitro. Wound‐healing assay for MCF7 and MDA-MB-231 and wound closure was monitored at 0, 24, and 48 h. Data in bar graphs are the means ± SD of three independent experiments. **P < 0.01; ***P < 0 .001 by Student t test. siRNA, small interfering RNA; RT-qPCR, Real Time Quantitative Polymerase Chain Reaction

In order to verify the results of above bioinformatics analysis, we further analyzed the FBXO1 protein levels in breast cancer and normal breast cell lines by Western blotting. FBXO1 was highly expressed in various breast cancer cell lines (MCF7, MDA-MB-231, MDA-MB-468, SK-BR3, T47D, HCC1954 and BT474), the expression levels were significantly higher than that in normal breast cell line (MCF-10A) (Fig. 8a). To examine the effect of FBXO1 in breast cancer cell lines, MCF7 and MDA-MB-231 were successfully transfected with si‐FBXO1 to knockdown expression of FBXO1 and verified by Real-time qPCR, Western-blot analysis and FAM-fluorescence detection (Fig. 8b–d). First of all, the CCK-8 assay was used to measure the proliferation of siRNA‐transfected cells. The MCF7 and MDA-MB-231 cell lines, treated with si‐FBXO1 #1 and #2, revealed the lower proliferative ability compared with the negative control groups (Fig. 8e). Besides, it turned out that colony formation in MCF7 and MDA-MB-231 cells was significantly reduced after FBXO1 depletion (Fig. 8f). Subsequently, we found that FBXO1 knockdown caused an apparent suppression of cell migration in MCF7 and MDA-MB-231cell lines (Fig. 8g, h). In conclusion, these results demonstrated that the knockdown of FBXO1 protein inhibited the proliferation and migration of breast cancer cells.

### Screening and functional analysis of 10 hub genes in Protein–Protein Interaction (PPI) network of FBXO1

**Fig. 9:**
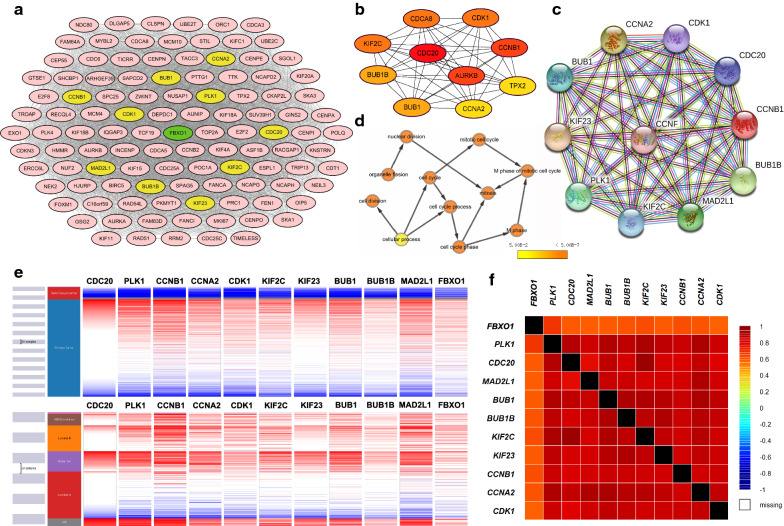
Protein–protein Interaction (PPI) Network and Correlative Analysis of FBXO1. **a** PPI network of FBXO1 and 108 co-expressed genes. The most significant modules and hub genes of the PPI network were analysed by Cytoscape software, which were marked in yellow. **b** The hub-genes were identified using cytoHubba tool kits in Cytoscape. **c** The PPI network of hub-genes were identified using STRING database. **d** The biological process analysis of hub-genes was performed using the BiNGO plug-in. P < 0.05 was considered to be a statistically significant difference. **e** The hierarchical clustering of hub-genes was constructed using UCSC online database. **f** The heat map of correlation between FBXO1 and hub-genes in BC patients analysed by bc-GenExMiner v4.0. Different color represents the percentage of correlationship. 1 stands for complete positive correlation and -1 stands for complete nagetive correlation

**Fig. 10:**
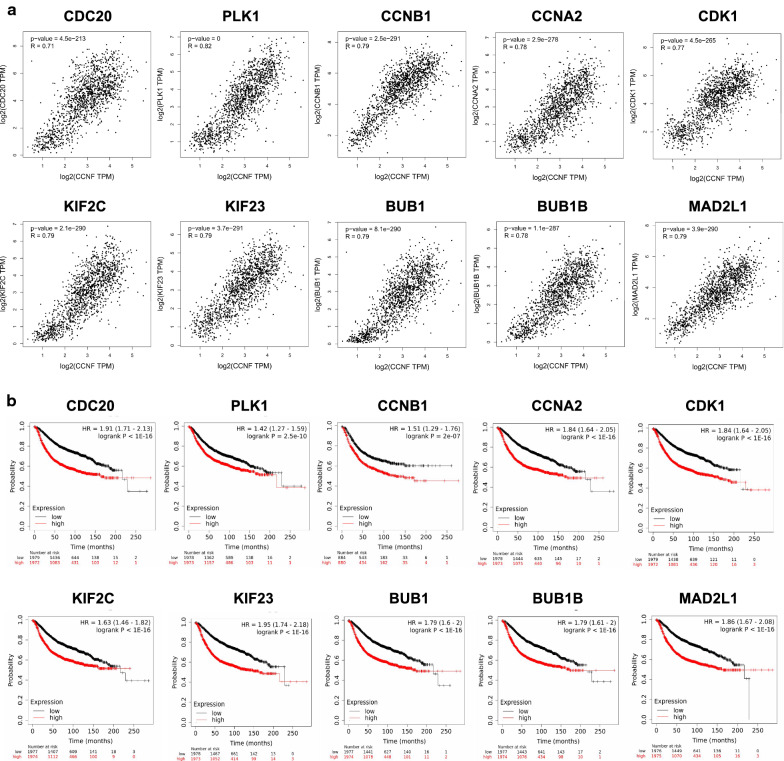
Correlative and Survial Analysis of Hub-genes. **a** Correlation between 10 hub-genes and FBXO1 mRNA expression determined by GEPIA database. **b** RFS survival analyses of hub-genes in BC. The results based on the KM plotter database indicated that all hub-genes were associated with poor prognosis in BC

Combined using the STRING database and Cytoscape software, we constructed a PPI network of the co-expressed 108 genes of FBXO1 and obtained the core gene modules. The top 10 genes included CDC20, PLK1, CCNB1, CCNA2, CDK1, KIF2C, KIF23, BUB1, BUB1B and MAD2L1, which were identified as potential hub genes according to the degree score generated by MCODE plug-in of Cytoscape (marked in yellow) (Fig. 9a). Meanwhile, according to the degree-rank score generated by CytoHubba plug-in, we got the similar top 10 hub nodes as Fig. 9a (Fig. 9b). Drawing support from STRING database, we further verified the strong correlation between FBXO1 and top 10 hub genes obtained from MCODE plug-in (Fig. 9c). In addition, BINGO plug-in showed the most significant biological process influenced by the hub genes, including cell cycle M phase, organelle fission and nuclear division, which suggesting that they probably play crucial roles in the tumor cell mitosis process (Fig. 9d). Hierarchical clustering of the 10 hub genes and FBXO1 was performed by UCSC Xena browser, indicating the consistent expression profile among these genes in overall and different subtypes of BC (Fig. 9e). The strong positive correlationship of transcriptional levels among FBXO1 and 10 hub genes in BC patients were also proved by heatmap from the bc-GenExMiner platform (Fig. 9f) and scatter diagram from the GEPIA dataset (Fig. 10a). To find more in-depth clinical significance of targeted genes, we investigated the Kaplan–Meier RFS survival curves of 10 hub genes in BC. The results displayed that high expression of total 10 hub genes predicted unfavorable prognosis in patients with BC (Fig. 10b). In conclusion, FBXO1 and CDC20, PLK1, CCNB1, CCNA2, CDK1, KIF2C, KIF23, BUB1, BUB1B, MAD2L1 may be tightly functional partners in regulating breast tumor cell cycle process and mediating poor prognosis of BC together.

## Discussion

Currently, the challenge of early detection and prediction of BC prognosis need to take better approaches. Searching of novel tumor-related molecular markers is in full swing. As new cancer biomarkers, FBXO factors dysregulation have been reported in many cancers like BC, HCC, gastric cancer, ovarian carcinoma and osteosarcoma. Although several FBXO family members have been confirmed to be related to BC, their distinct molecular mechanism remains to be explored. We found the mRNA expression of 10 FBXO members was remarkable altered and correlated with tumor clinical stage, pathological grade and the prognosis of BC. In this article, we firstly probe into the transcription levels and prognostic values (RFS, OS, DMFS and PPS) of 10 FBXO family members (1,2, 5, 6, 16, 17, 22, 28, 31 and 45) in BC**.** We hope that our findings will contribute to available knowledge, enhance the accuracy of diagnosis and prognosis for BC patients.

FBXO1 has been identified as an expected tumor suppressor which can induce G2 phase arrest, impede the initiation of mitosis when it’s overexpressed in cells [37]. Previous studies have demonstrated that down regulation of FBXO1 can accelerate tumor growth, which is related to advanced tumor stage, poor survival rate in hepatocellular carcinoma (HCC) [38]. As far as we know, the function of FBXO1 involved in tumorigenesis and development are not fully elucidated in BC. In our study, we demonstrate that FBXO1 was high-expressed in all subtypes of BC, and similar results were confirmed by immunohistochemistry. It was an independent poor prognostic factor of RFS, OS and DMFS in BC by Kaplan–Meier Plotter. Besides, targeting FBXO1 may be a promising strategy for therapeutic intervention against hormone receptor-positive types of BC because high expression of FBXO1 means shorter RFS, OS and DMFS in luminal A subtypes. Most importantly, we excavated the top 10 height-correlation oncogene cluster of FBXO1, which were CDC20, PLK1, CCNB1, CCNA2, CDK1, KIF2C, KIF23, BUB1, BUB1B and MAD2L1. They might interact and jointly mediate the development of BC. To explore the interaction mechanism between FBXO1 and these oncogenes in cell cycle is one novel direction in future research work.

We speculated that FBXO2, FBXO6, FBXO16 and FBXO17 were potential favorable prognostic factors for BC and all of them were correlated to clinicopathological staging. Previous study literature has indicated that high expression of FBXO2 promotes the proliferation and migration of gastric cancer cells and which is related to shorter OS of patients. FBXO2 may be a novel clinical target for gastric cancer because low FBXO2 expression can increase the mRNA levels of E-cadherin but reduce the expression of N-cadherin in gastric cancer cell. Down-regulating of FBXO2 inhibits the migration of gastric cancer by reducing EMT [39]. The other study shows FBXO2 is significantly up-regulated in osteosarcoma, which may modulate STAT3 signaling to regulate proliferation and tumorigenicity of osteosarcoma cells [40]. Interestingly, we demonstrated that FBXO2 was down-regulated generally in BC, overexpression of FBXO2 stands for better RFS in Luminal B and HER2 types BRCA, while the expression was correlated with tumor stage in patients with BC. It seemed consistent with the role of FBXO2 as a tumor suppressor. It has been proved that low levels of FBXO6 and consequent impairment of replication stress-induced Chk1 degradation are associated with resistance to camptothecin of BC [13], the similar results about drug-resistance have been confirmed in small cell lung cancer by Cai et al. [41]. We also found that up-regulated FBXO6 represented superior RFS in HER2 and TNBC types of patients. It was highly expressed in all subtypes of tumors and closely related to different clinical stages. Thus, FBXO6 may be an excellent prognostic marker and therapeutic target to overcome the drug-resistance of chemotherapy agents in BC patients. In one sense, FBXO16 is a putative tumor suppressor that suppresses the growth, migration and invasion of cancer cells. It interacts physically with the C-terminal domain of β-catenin and promotes its lysine 48-linked polyubiquitination and it can inhibit EMT by attenuating the level of β-catenin in BC cells [42]. This was consistent with our conclusion, the mRNA levels of FBXO16 were especially high in Luminal B subtypes and which was associated with better prognosis. Therefore, FBXO16 may be a putative tumor suppressor. In general, it has been proved that FBXO17 is overexpressed in many kinds of tumors, like glioma [15], HCC [43], lung adenocarcinoma [16] and esophageal squamous cell carcinoma [44]. It may affect multiple cellular signaling pathways like Wnt/β-catenin [43] or PI3K-Akt [16]. Overexpression of FBXO17 is significantly associated with poor prognosis of these cancer patients. The role of FBXO17 in BC has not been elucidated. We put forward a new viewpoint of FXBO17 by analyzing tumor databases. FBXO17 was significantly down-regulated in all subtypes of BRCA, and overexpression did not mediate the adverse outcomes of BC patients. By contrast, high mRNA expression of FBXO17 indicated better RFS outcomes for BC patients. In our point of view, focus on researching the functions of FBXO17 may promote the advances of molecular mechanism of BC.

As for FBXO5, FBXO22, FBXO28, FBXO31 and FBXO45, they may be the independent poor prognostic factors of BC and the expression levels of which were closely related to different tumor stages. Significant overexpression of FBXO5 has been detected in mixed endometrioid/clear ovarian cell tumors but absent in ovarian tumors with mixed serous/clear cell histology [45]. Besides, FBXO5 has been proved positively correlated with stage and poor outcome in HCC [46]. Also, we got the similar results in BC. In luminal A type of BC, overexpression of FBXO5 stands for poor RFS, OS, DMFS and PPS, and poor RFS in luminal B type of patients. The mechanism of action of FBXO5 was related to poor prognosis in BC, which was worthy to make a profound study in the future. Overexpression of FBXO22 has been reported to suppress the Bach1‐driven metastasis of lung adenocarcinoma [17] and promote nuclear tumor suppressive factor PTEN downregulation to play a tumor-promoting role in colorectal cancer [47]. However, Yoshikazu et al. have showed that low levels of FBXO22 in HER2-negative BC predict a poorer outcome with high hazard ratios, independently of other markers such as Ki-67 and lymphnode metastasis status [48]. FBXO22 targets cellular HDM2 for ubiquitin-dependent degradation and low expression of FBXO22 is correlated with worse survival and high HDM2 expression in human BC in Bai’s study [49]. This is a controversial molecular biomarker. In our report, we found that FBXO22 had high expression levels in all subtypes, especially in HER2 type of BC, which symbolized worse OS in HER2 + patients. High expression level of FBXO28 is associated with worse BC outcomes through non-proteolytic ubiquitination of MYC143 to stimulate cancer cell transcription [20]. We conjectured FBXO28 was a potential adverse prognostic factor in luminal B and triple-negative of BC by Kaplan–Meier plotter. High-regulation of FBXO31 inhibits the proliferation and colony formation of breast tumor cells by mediating ubiquitination and degradation of specific substrates, and then inhibits cancer progression [50, 51]. Nevertheless, we obtained interesting conclusion of FBXO31. Although it was down-regulated in all subtypes of BC, overexpression of which represented poor prognosis in Luminal A, B and HER2 types. Maybe FBXO31 didn’t function as a tumor suppressor, the mechanism of action in BC still need to further explore. Some studies have uncovered that FBXO45 may have important roles in tumorigenesis and progression. The gastric cancer patients with low FBXO45 expression exhibits poorer survival outcomes [52]. However, the mechanism of FBXO45 in BC remains to explore. There is evidence that FBXO45 mediates ubiquitylation and proteasomal degradation of prostate apoptosis response protein 4, a tumor suppressor protein located in the cytoplasm, to develop a critical role in survival and activity of tumor cells [53]. In our study, we draw the conclusion that FBXO45 had a high expression levels in all subtypes of BC. It was also highly correlated with tumor patients with different pathological stages. In luminal A and B types of BC groups, FBXO45 showed poorer RFS and OS clinical outcomes. Thus, FBXO45 leads to poor prognosis and may be a novel therapeutic target for BC treatment.

## Conclusion

In summary, our research work indicates that FBXO2, FBXO6, FBXO16 and FBXO17 may be the potential favorable prognostic factors of BC patients. FBXO1, FBXO5, FBXO22, FBXO28, FBXO31 and FBXO45 are significantly correlated with worse clinical survival outcomes. Based on the above findings, it’s expected that FBXO1 could act as the most promising prognostic biomarker and therapeutic target for BC. These molecules shed more light on the complexity and heterogeneity of BC biological properties, and further mechanistic studies are needed to validate our findings and to promote clinical application of FBXOs in BC. We hope our research findings could contribute to a better understanding of the pathological mechanism of BC and assist in searching for effective cancer therapeutic targets to improve the BC survival and prognostic accuracy.

## Supplementary Information

**Additional file 1: Figure S1.** The Comparation of FBXOs Expression Situation in Various Tumor and Normal Samples across TCGA Datasets Using Oncomine Databases. Red, over-expression; Blue, down-regulated expression.

**Additional file 2: Figure S2.** The Comparation of FBXOs Expression Situation in Various Tumor and Normal Samples across TCGA Datasets Using UALCAN Datasets. Red, tumor samples; Blue, normal samples.

**Additional file 3: Figure S3.** The prognostic values of FXBO family members in different subtypes of BC patients. The survival curves comparing BC patients with high (red) and low (black) FBXO expression levels were plotted using the Kaplan-Meier Plotter. DFS, disease-free survival rate; OS, the overall survival rate; DMFS, distance metastasis free survival; PPS, post progression survival; The threshold P-value is less-than 0.05.

**Additional file 4: Table S1.** Summary of clinical data of breast cancer patients whose samples were used in Immunohistochemical Staining.

**Additional file 5: Figure S4.** KEGG analysis of cell cycle pathway regulated by the FBXO1 and co-expression genes alteration in BC are shown in DAVID database. Altered genes of the pathway marked in red.

## Data Availability

Source data of this study were derived from the public repositories, as indicated in the section of “Materials and Methods” of the manuscript. And all data that support the findings of this study are available from the corresponding author upon reasonable request.

## Abbreviations

BC: Breast cancer
FBXO: F-box only with uncharacterized domains
EMT: Epithelial-mesenchymal transition
ER: Estrogen receptor
PR: Progesterone receptor
HER2: Human epidermal growth factor receptor 2
TNBC: Triple-negative statues breast cancer
BRCA: Breast invasive carcinoma
HCC: Hepatocellular carcinoma
TCGA: The Cancer Genome Atlas
TPM: Transcripts per million
HPA: The Human Protein Altas
IHC: Immunohistochemistry
DFS: Disease-free survival rate
OS: The overall survival rate
DMFS: Distance metastasis free survival rate
PPS: Post progression survival rate
GO: Gene Ontology
KEGG: Kyoto Encyclopedia of Genes and Genomes
BP: Biological process
CC: Cell component
MF: Molecular function
PPI: Protein–Protein Interaction
