# Supplementary material for: Systematic analysis of the expression and prognosis relevance of FBXO family reveals the significance of FBXO1 in human breast cancer
Source: Cancer Cell Int. 2021 Feb 23;21:130. doi: 10.1186/s12935-021-01833-y (PMC7903729; doi:10.1186/s12935-021-01833-y)

Figure S1

Analysis Type by Cancer	FBXO1		FBXO2		FBXO5		FBXO6		FBXO16		FBXO17		FBXO22		FBXO28		FBXO31		FBXO45	
	Cancer vs. Normal		Cancer vs. Normal		Cancer vs. Normal		Cancer vs. Normal		Cancer vs. Normal		Cancer vs. Normal		Cancer vs. Normal		Cancer vs. Normal		Cancer vs. Normal		Cancer vs. Normal	
Bladder Cancer	4														1					
Brain and CNS Cancer		1	1	8	6	1				1	1			1				1		2
Breast Cancer	11			3	3	2	5		3			3	1	1	3				2	
Cervical Cancer	2				3								1		1				1	
Colorectal Cancer	8		1	1	9				2				2		1				4	
Esophageal Cancer	1	1		1	1				1							1				
Gastric Cancer	5				1										1		3		1	
Head and Neck Cancer			1		1		4			1			1				2		5	
Kidney Cancer		1	1	1			1				1		3				1		1	
Leukemia		4				3		1								2	4	2		1
Liver Cancer																1				
Lung Cancer	7				2				1			1	3						5	
Lymphoma		1			1		9						3		2	3		1		1
Melanoma				1							2					1	1			
Myeloma													1		2					
Other Cancer	5		1	1	5					3		1	1		5	1		7	3	
Ovarian Cancer	1		1		1				1							1				
Pancreatic Cancer				2	1															
Prostate Cancer		1										3								
Sarcoma	4	1		4	6						2	1	2					1		
Significant Unique Analyses	47	10	6	21	39	6	19	1	8	5	6	9	17	2	16	9	11	12	22	4
Total Unique Analyses	358		269		270		200		175		245		278		327		291		205	

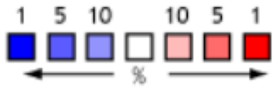

Supplement: Supplementary file 1 — Additional file 1: Figure S1. The Comparation of FBXOs Expression Situation in Various Tumor and Normal Samples across TCGA Datasets Using Oncomine Databases. Red, over-expression; Blue, down-regulated expression. [file 12935_2021_1833_MOESM1_ESM.pdf]
