# Supplementary figures and images for: Systematic analysis of the expression and prognosis relevance of FBXO family reveals the significance of FBXO1 in human breast cancer
Source: Cancer Cell Int. 2021 Feb 23;21:130. doi: 10.1186/s12935-021-01833-y (PMC7903729; doi:10.1186/s12935-021-01833-y)

Figure S2

FBXO1

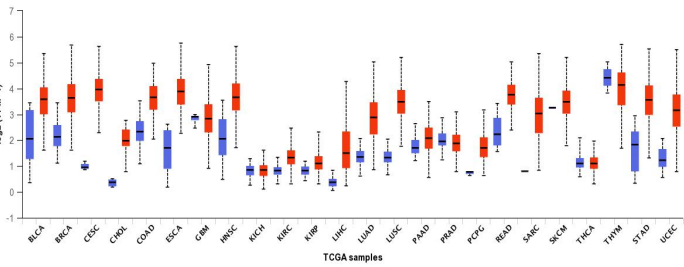

FBXO2

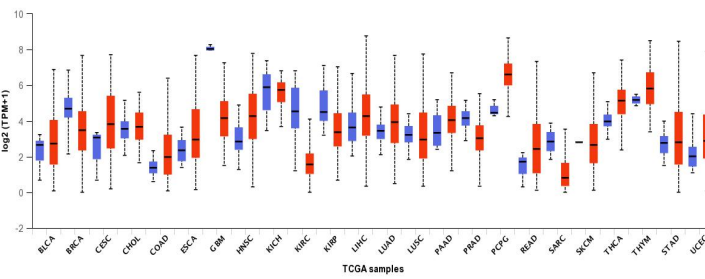

FBXO5

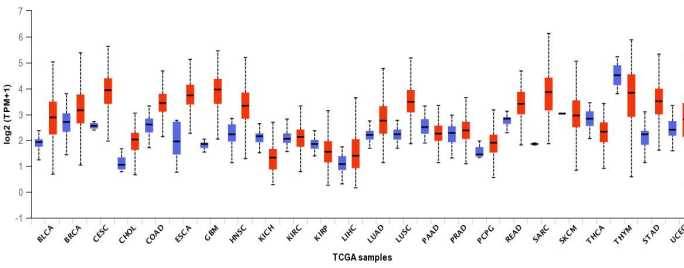

FBXO6

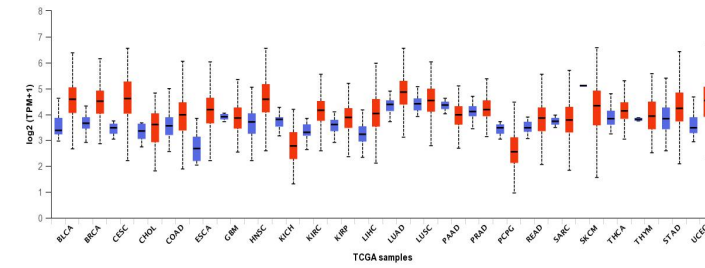

FBXO16

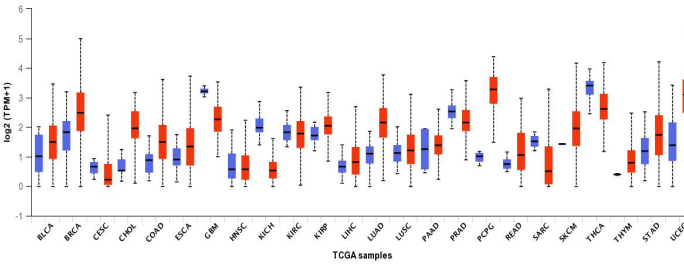

FBXO17

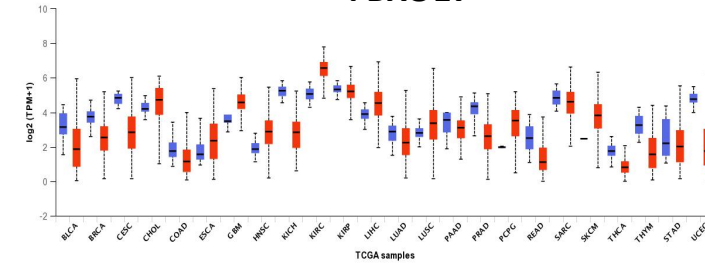

FBXO22

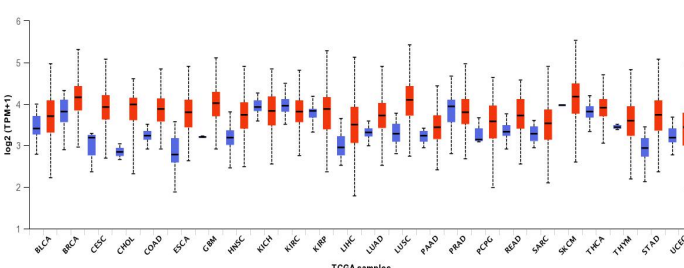

FBXO28

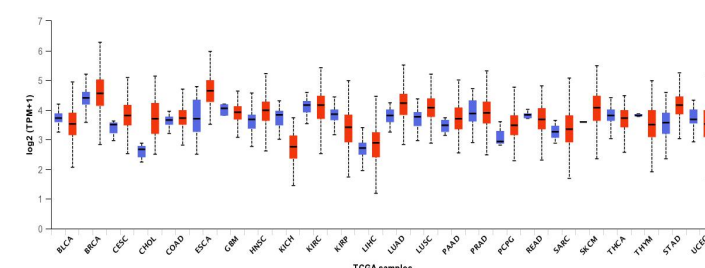

FBXO31

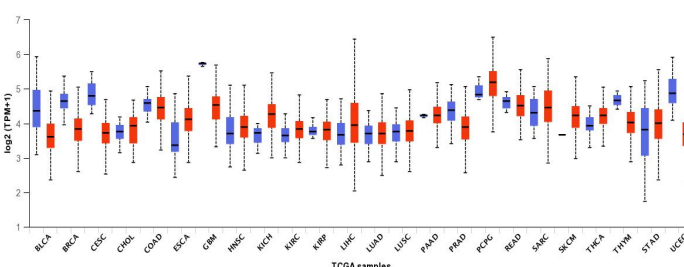

FBXO45

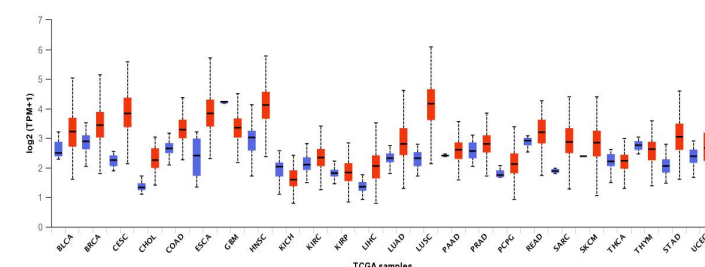

Supplement: Supplementary file 2 — Additional file 2: Figure S2. The Comparation of FBXOs Expression Situation in Various Tumor and Normal Samples across TCGA Datasets Using UALCAN Datasets. Red, tumor samples; Blue, normal samples. [file 12935_2021_1833_MOESM2_ESM.pdf]

Figure S3

FBXO1

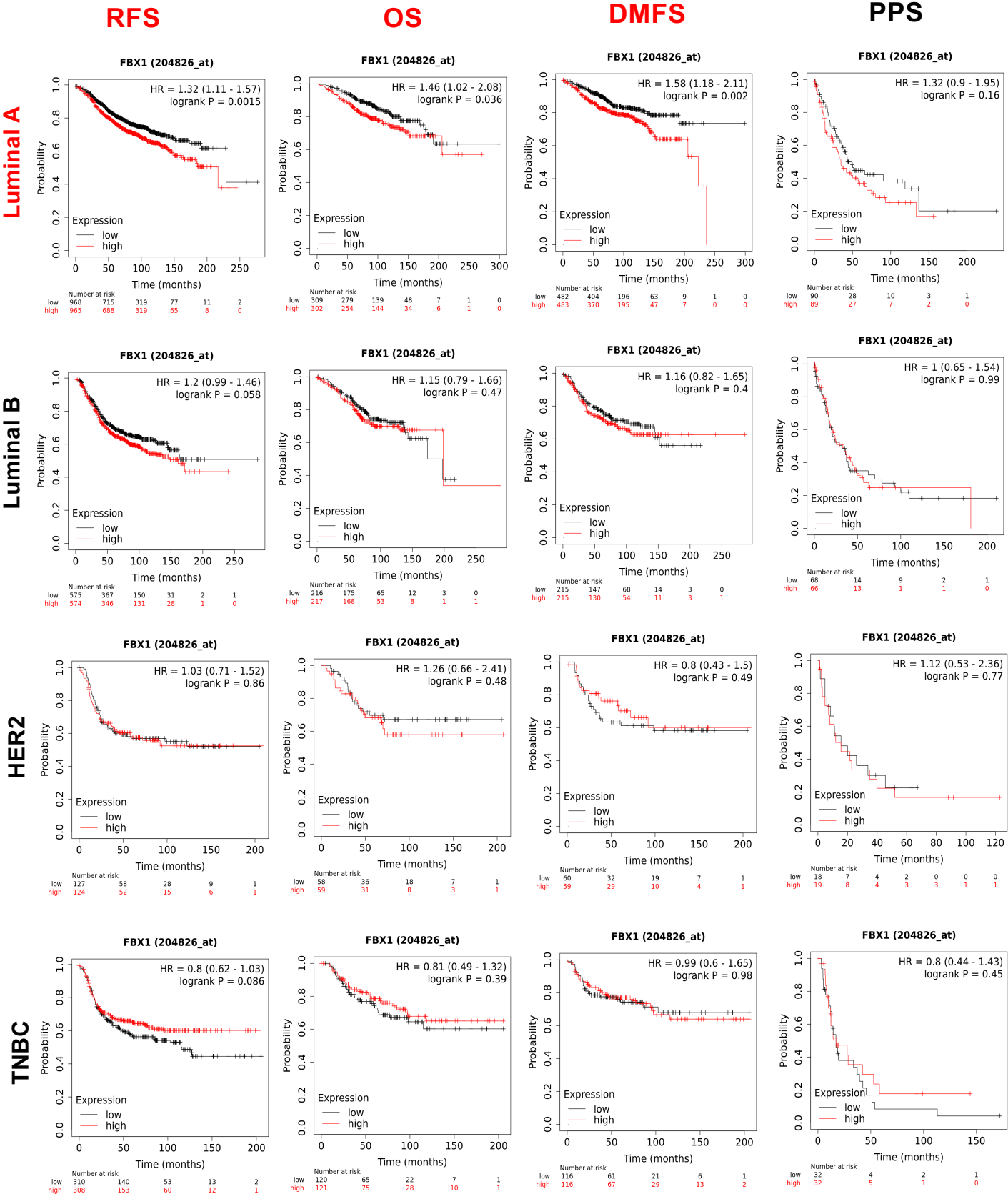

Supplement: Supplementary file 3 — Additional file 3: Figure S3. The prognostic values of FXBO family members in different subtypes of BC patients. The survival curves comparing BC patients with high (red) and low (black) FBXO expression levels were plotted using the Kaplan-Meier Plotter. DFS, disease-free survival rate; OS, the overall survival rate; DMFS, distance metastasis free survival; PPS, post progression survival; The threshold P-value is less-than 0.05. [file 12935_2021_1833_MOESM3_ESM.zip › Figure S3-1.pdf]

FBXO45

RFS

OS

DMFS

PPS

Luminal A

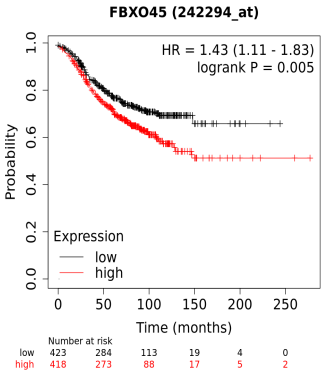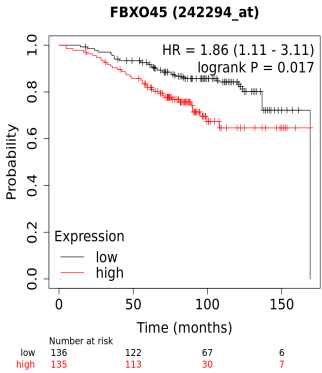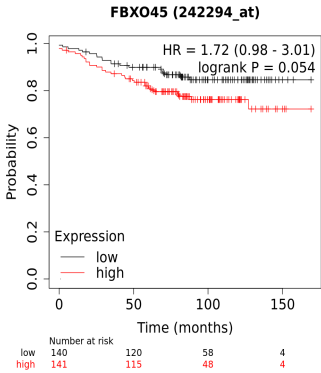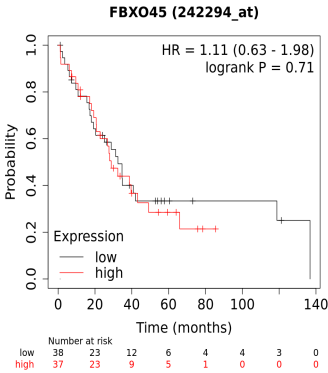

Luminal B

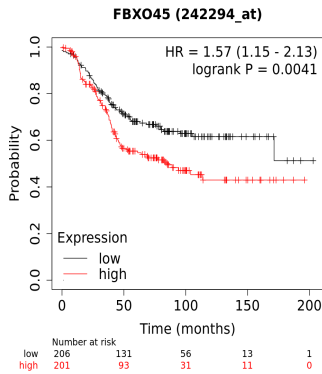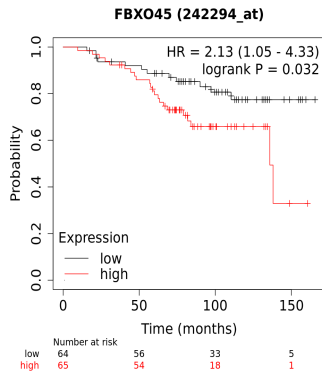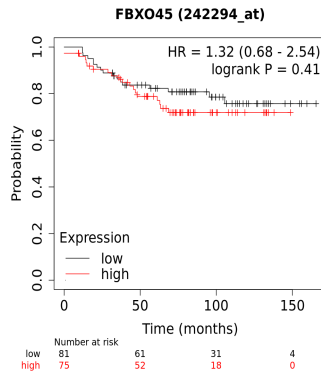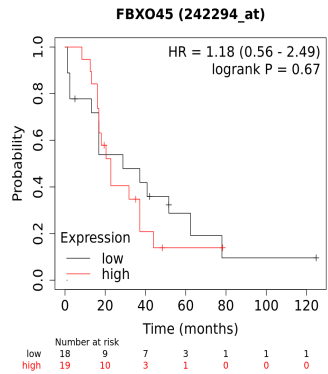

HER2

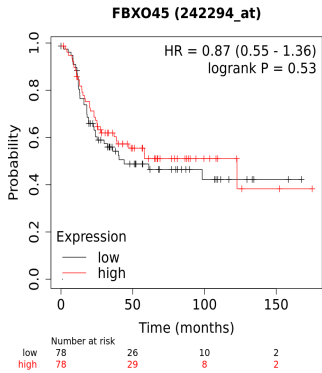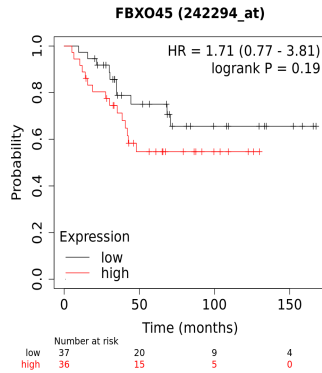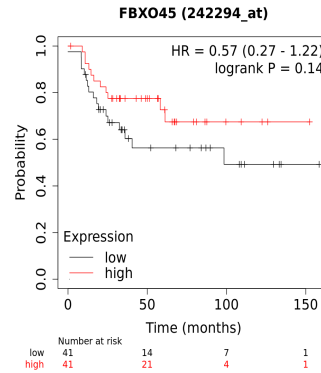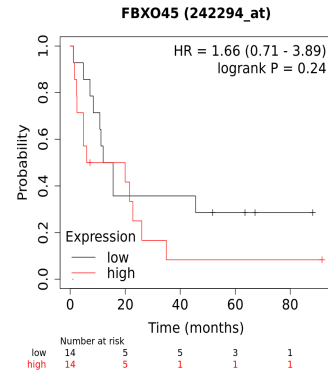

TNBC

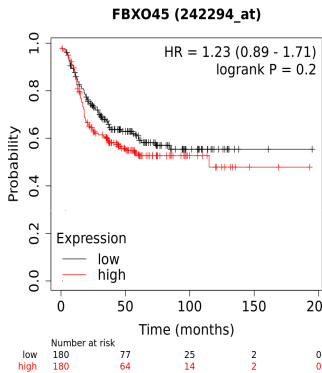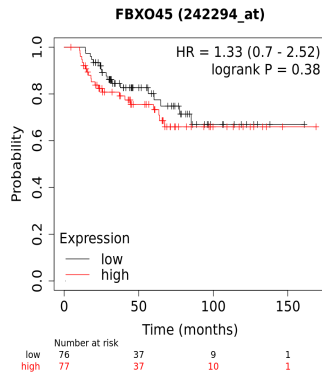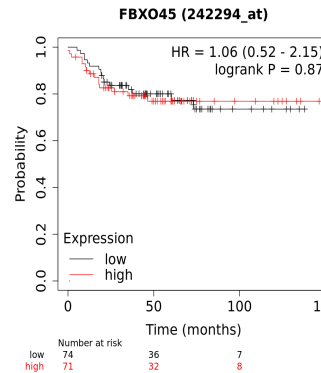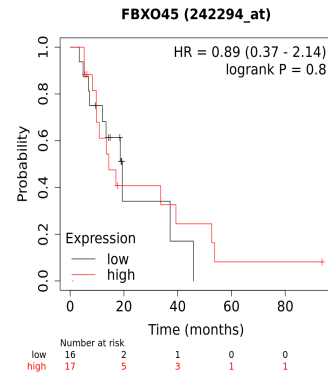

Supplement: Supplementary file 3 — Additional file 3: Figure S3. The prognostic values of FXBO family members in different subtypes of BC patients. The survival curves comparing BC patients with high (red) and low (black) FBXO expression levels were plotted using the Kaplan-Meier Plotter. DFS, disease-free survival rate; OS, the overall survival rate; DMFS, distance metastasis free survival; PPS, post progression survival; The threshold P-value is less-than 0.05. [file 12935_2021_1833_MOESM3_ESM.zip › Figure S3-10.pdf]

FBXO2

RFS

OS

DMFS

PPS

Luminal A

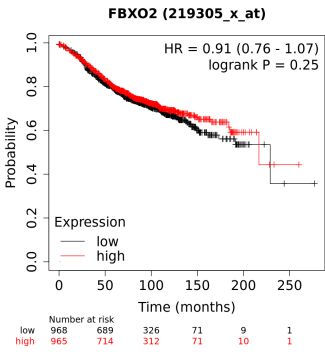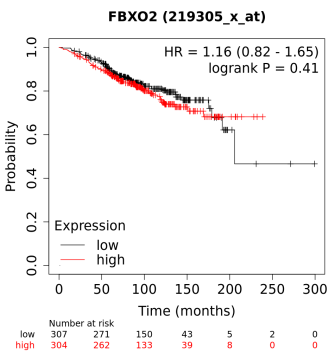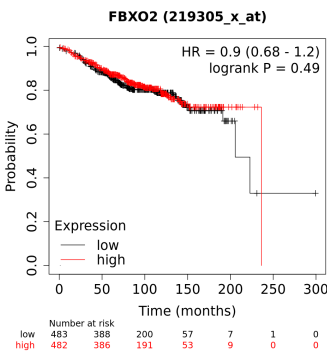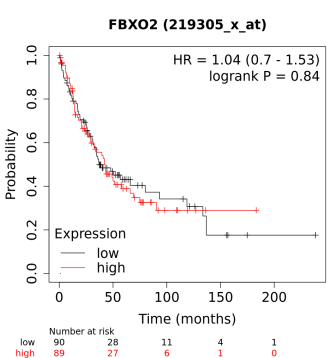

Luminal B

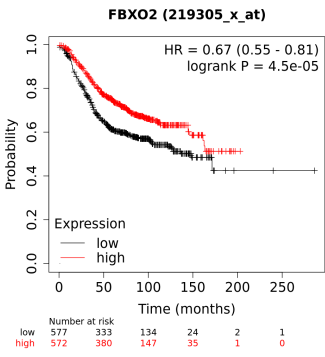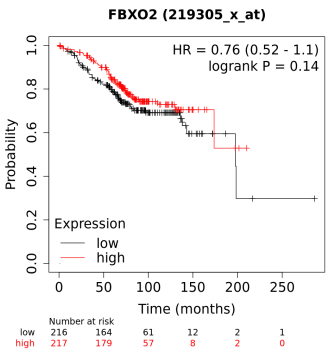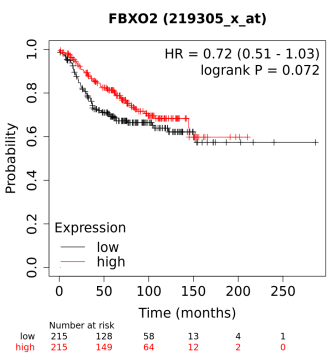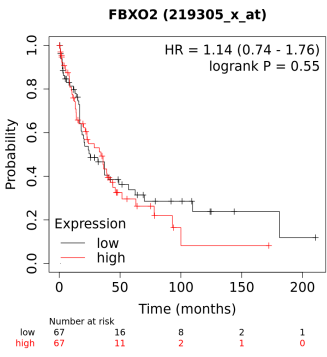

HER2

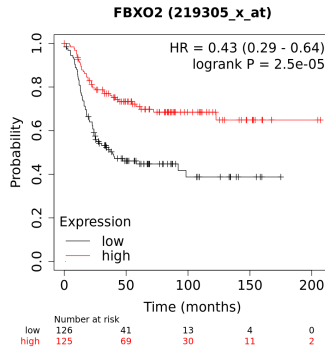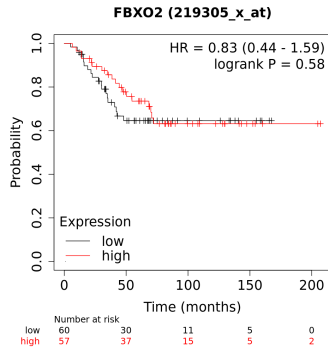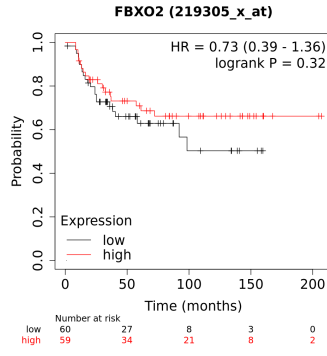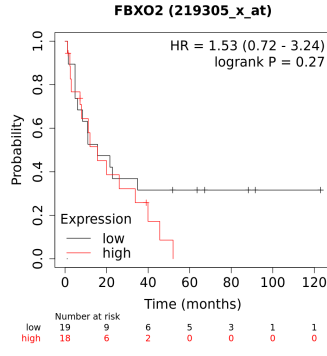

TNBC

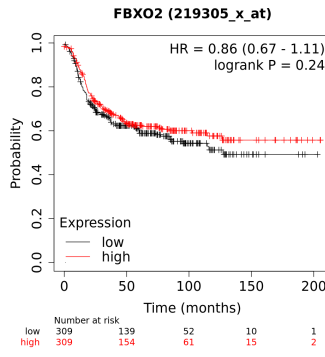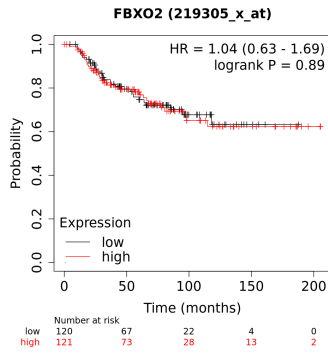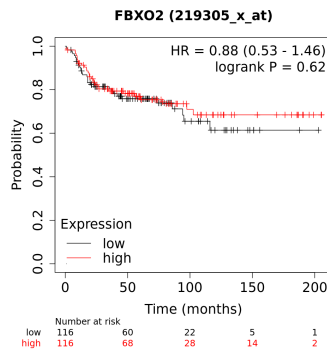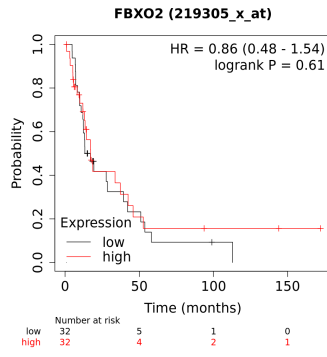

Supplement: Supplementary file 3 — Additional file 3: Figure S3. The prognostic values of FXBO family members in different subtypes of BC patients. The survival curves comparing BC patients with high (red) and low (black) FBXO expression levels were plotted using the Kaplan-Meier Plotter. DFS, disease-free survival rate; OS, the overall survival rate; DMFS, distance metastasis free survival; PPS, post progression survival; The threshold P-value is less-than 0.05. [file 12935_2021_1833_MOESM3_ESM.zip › Figure S3-2.pdf]

FBXO5

RFS

OS

DMFS

PPS

Luminal A

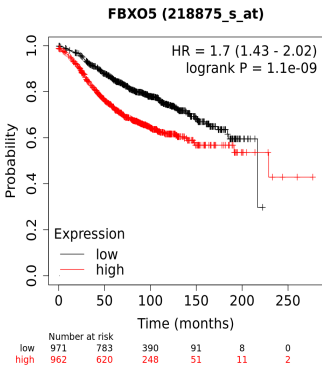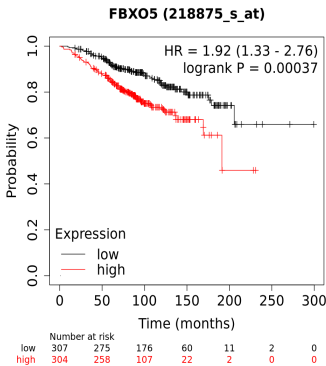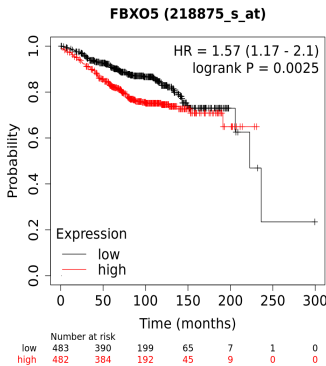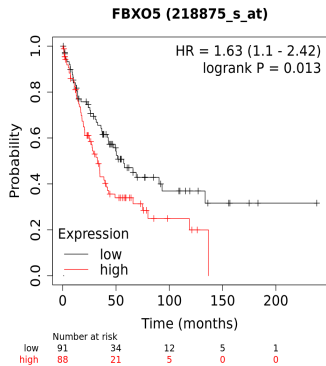

Luminal B

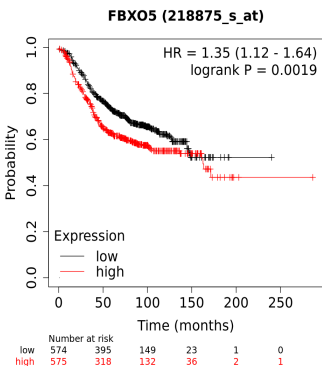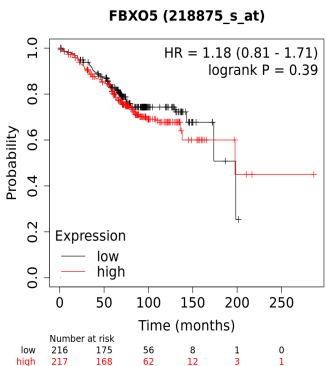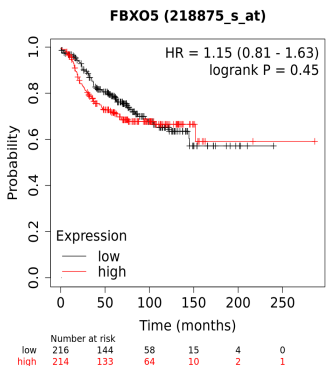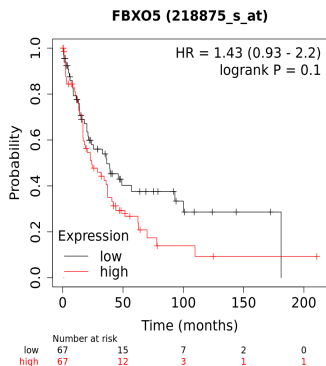

HER2

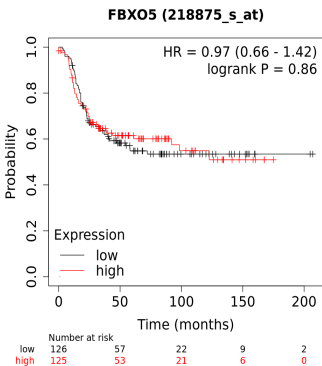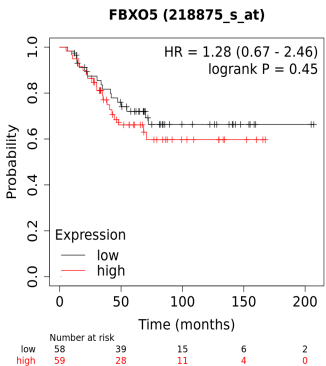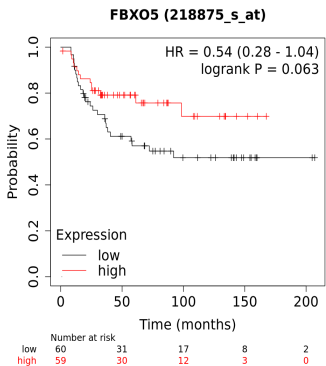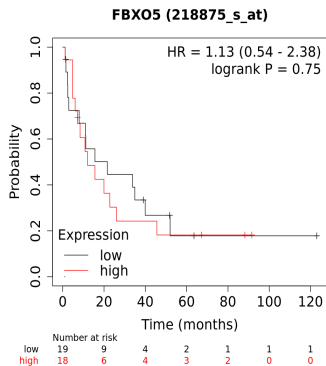

TNBC

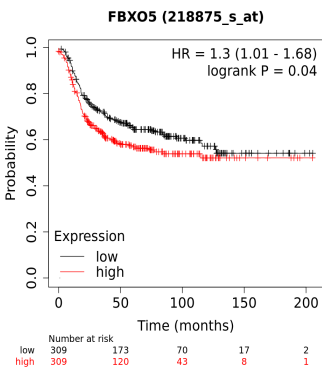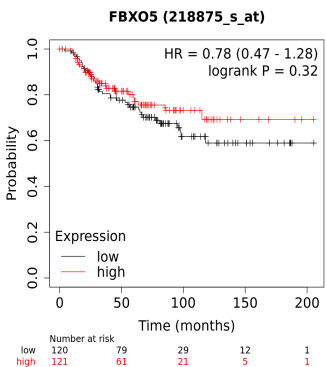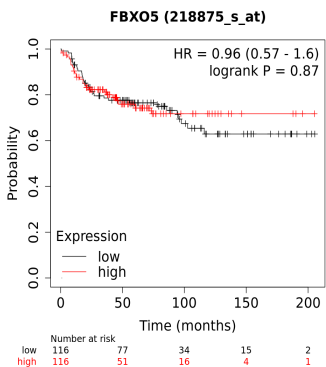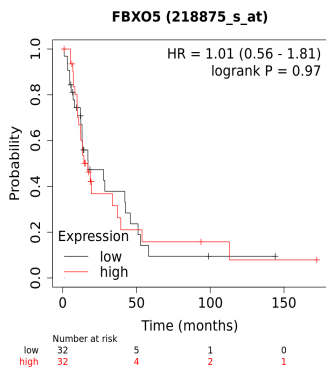

Supplement: Supplementary file 3 — Additional file 3: Figure S3. The prognostic values of FXBO family members in different subtypes of BC patients. The survival curves comparing BC patients with high (red) and low (black) FBXO expression levels were plotted using the Kaplan-Meier Plotter. DFS, disease-free survival rate; OS, the overall survival rate; DMFS, distance metastasis free survival; PPS, post progression survival; The threshold P-value is less-than 0.05. [file 12935_2021_1833_MOESM3_ESM.zip › Figure S3-3.pdf]

# FBXO6

RFS

OS

DMFS

PPS

Luminal A

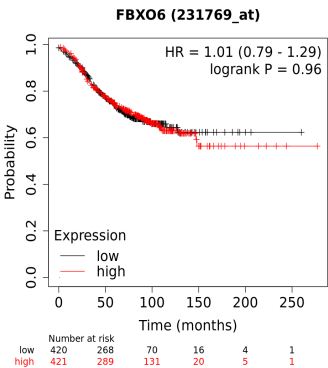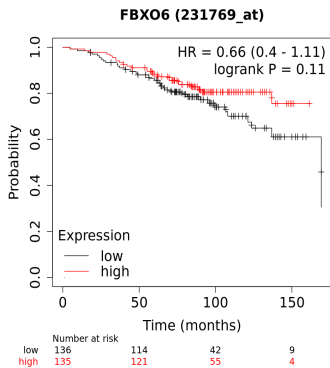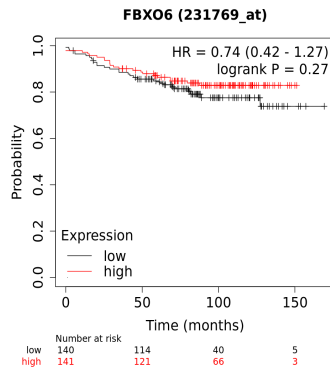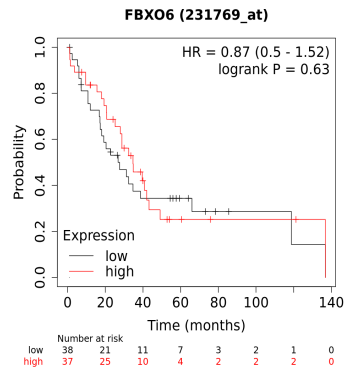

Luminal B

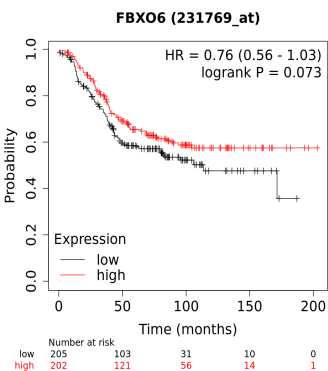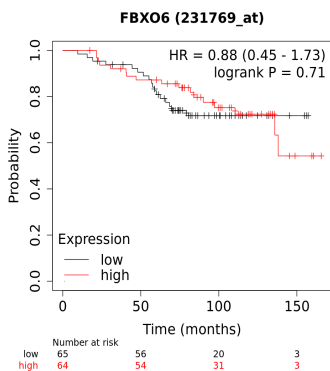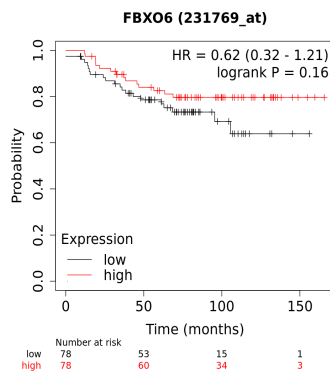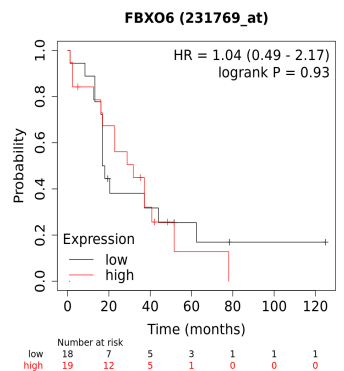

HER2

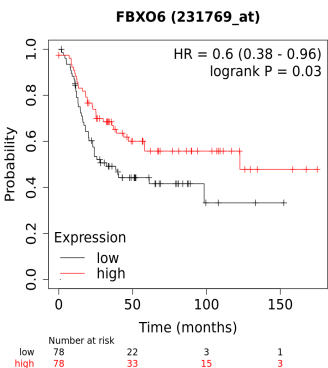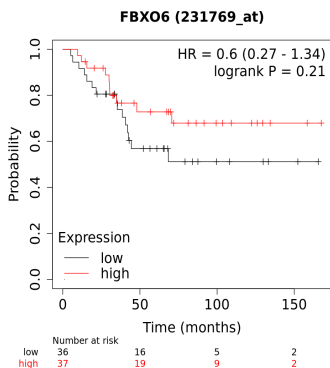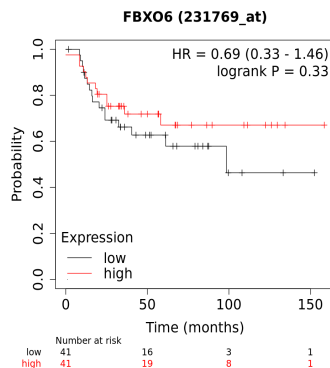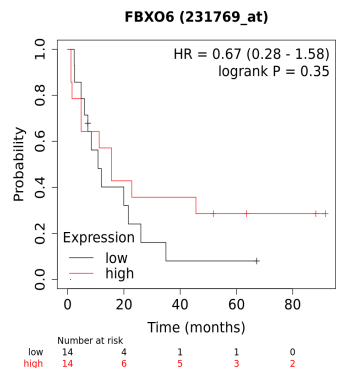

TNBC

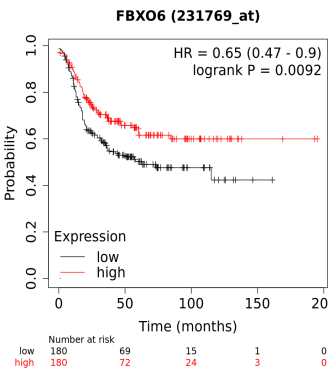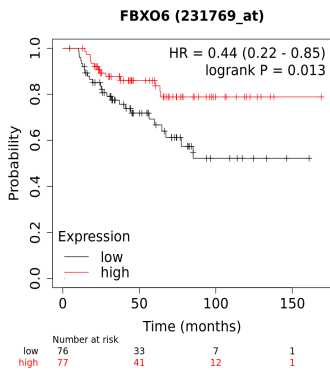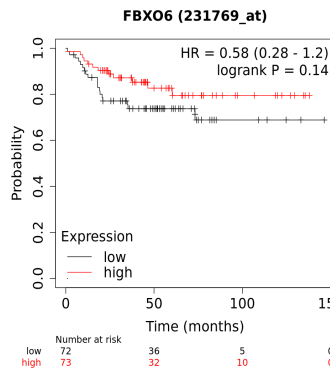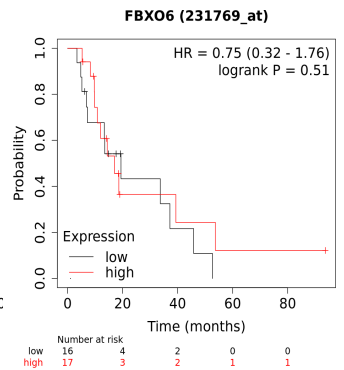

Supplement: Supplementary file 3 — Additional file 3: Figure S3. The prognostic values of FXBO family members in different subtypes of BC patients. The survival curves comparing BC patients with high (red) and low (black) FBXO expression levels were plotted using the Kaplan-Meier Plotter. DFS, disease-free survival rate; OS, the overall survival rate; DMFS, distance metastasis free survival; PPS, post progression survival; The threshold P-value is less-than 0.05. [file 12935_2021_1833_MOESM3_ESM.zip › Figure S3-4.pdf]

FBXO16

RFS

OS

DMFS

PPS

Luminal A

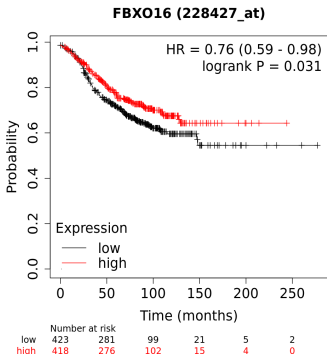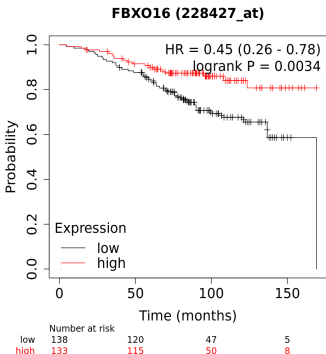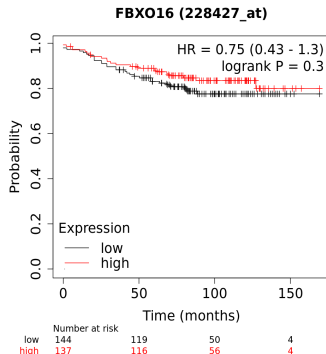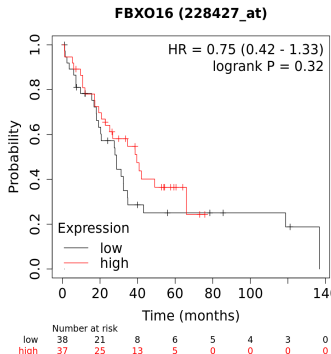

Luminal B

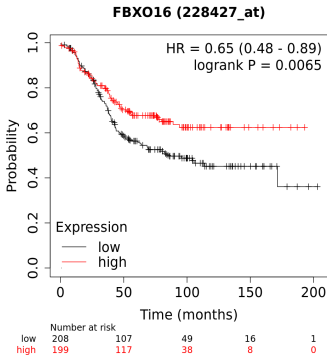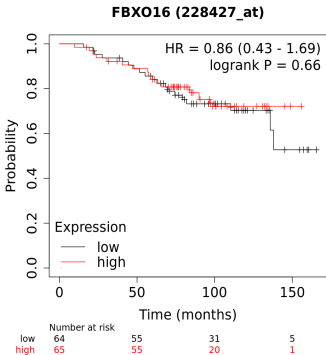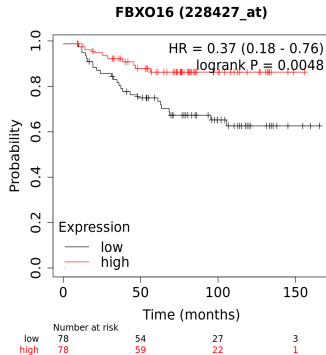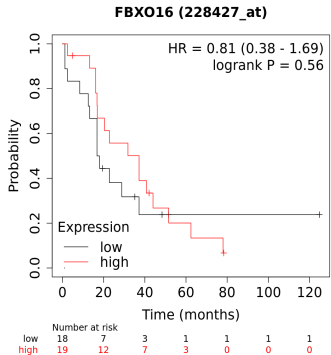

HER2

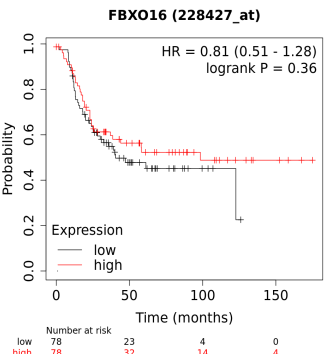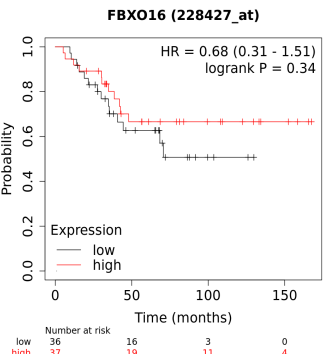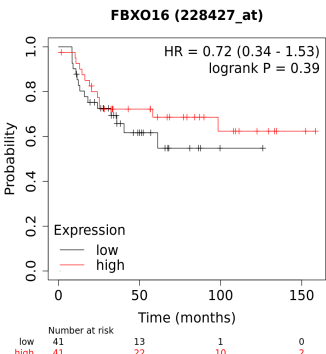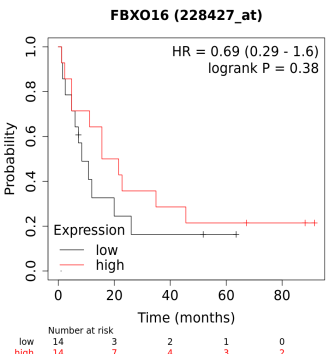

TNBC

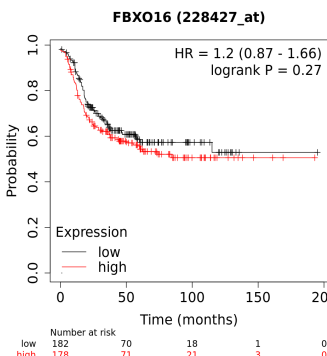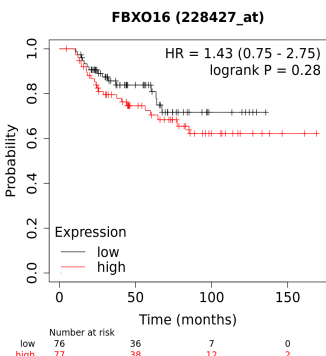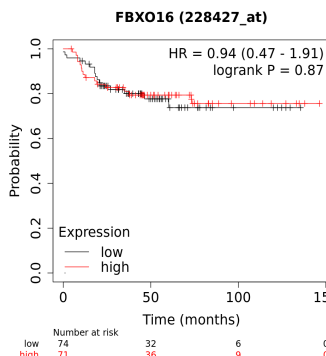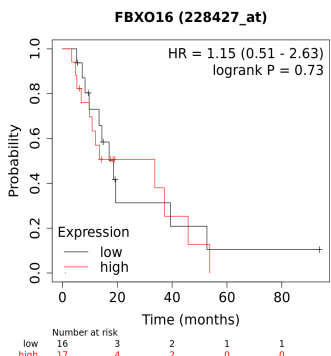

Supplement: Supplementary file 3 — Additional file 3: Figure S3. The prognostic values of FXBO family members in different subtypes of BC patients. The survival curves comparing BC patients with high (red) and low (black) FBXO expression levels were plotted using the Kaplan-Meier Plotter. DFS, disease-free survival rate; OS, the overall survival rate; DMFS, distance metastasis free survival; PPS, post progression survival; The threshold P-value is less-than 0.05. [file 12935_2021_1833_MOESM3_ESM.zip › Figure S3-5.pdf]

# FBXO17

RFS

OS

DMFS

PPS

Luminal A

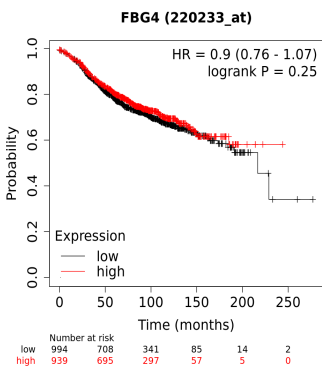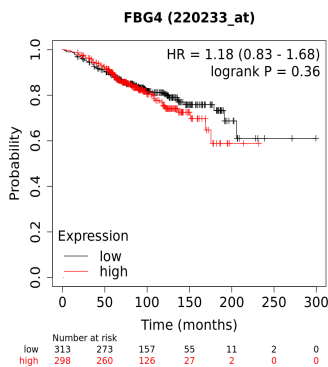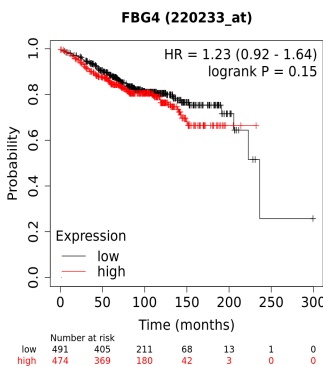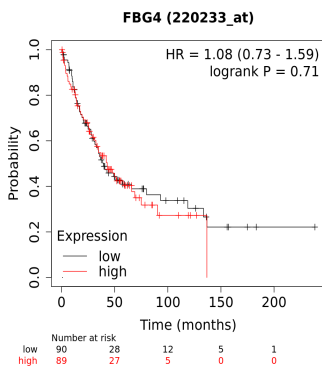

Luminal B

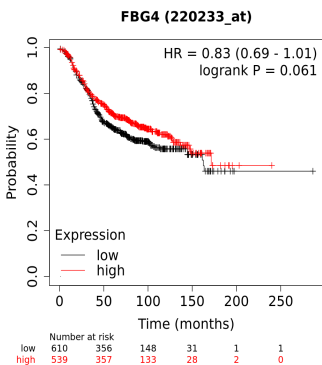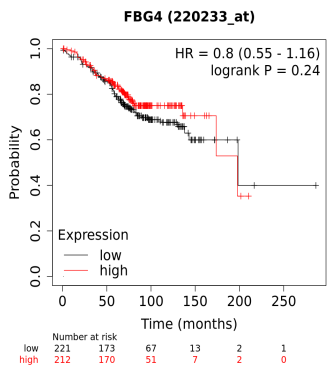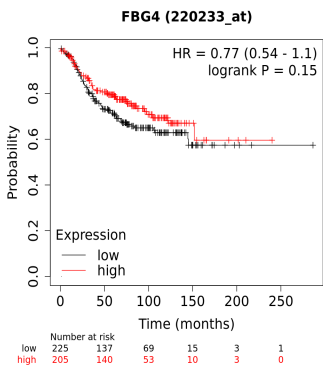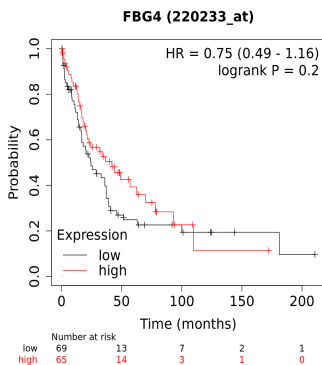

HER2

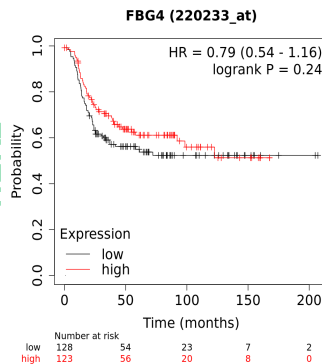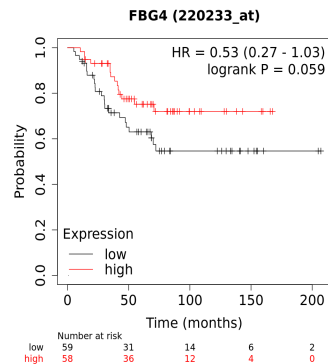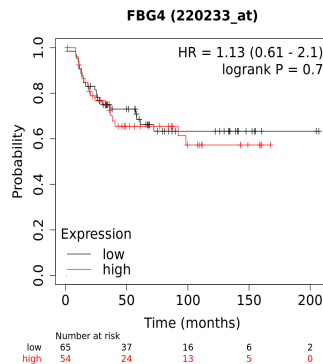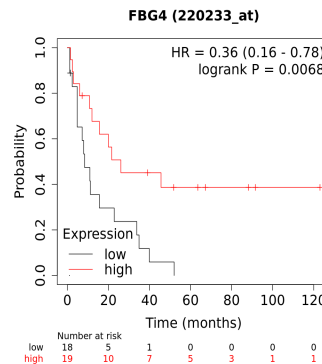

TNBC

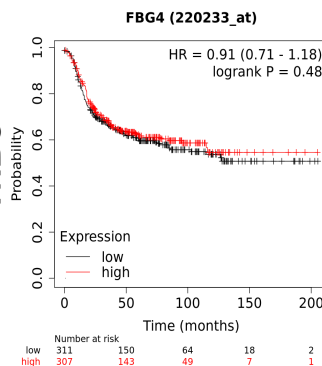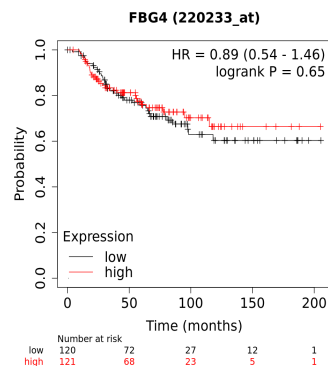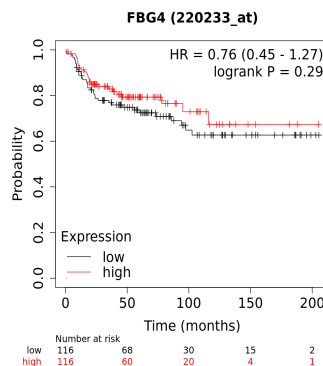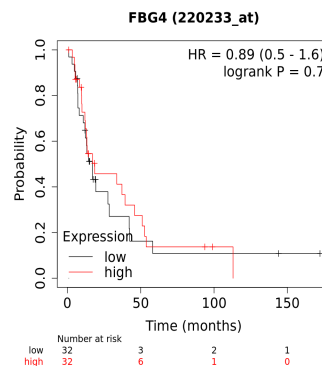

Supplement: Supplementary file 3 — Additional file 3: Figure S3. The prognostic values of FXBO family members in different subtypes of BC patients. The survival curves comparing BC patients with high (red) and low (black) FBXO expression levels were plotted using the Kaplan-Meier Plotter. DFS, disease-free survival rate; OS, the overall survival rate; DMFS, distance metastasis free survival; PPS, post progression survival; The threshold P-value is less-than 0.05. [file 12935_2021_1833_MOESM3_ESM.zip › Figure S3-6.pdf]

FBXO28

RFS

OS

DMFS

PPS

Luminal A

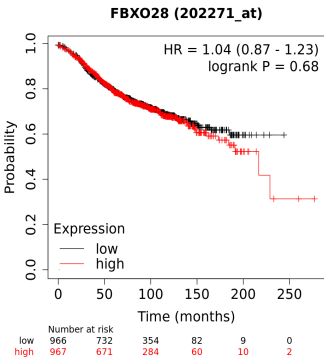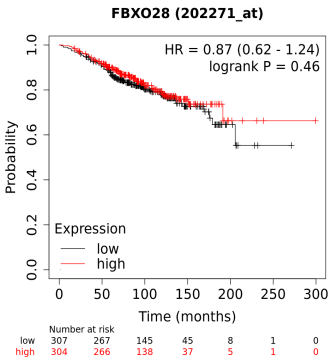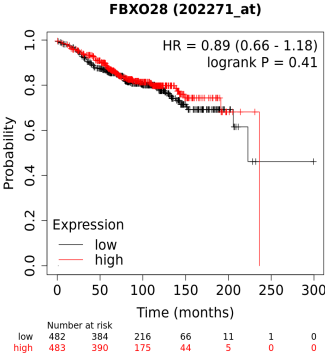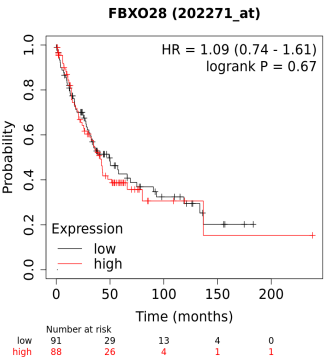

Luminal B

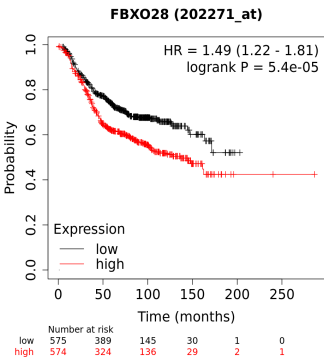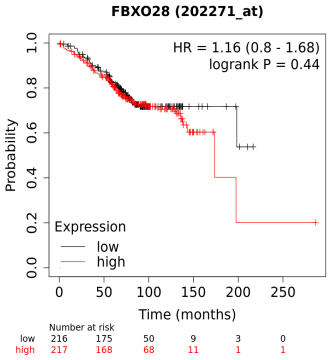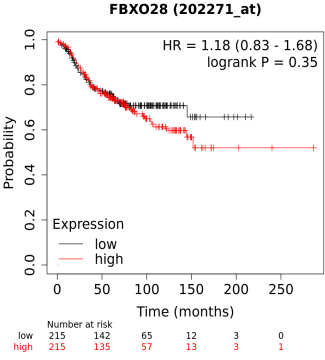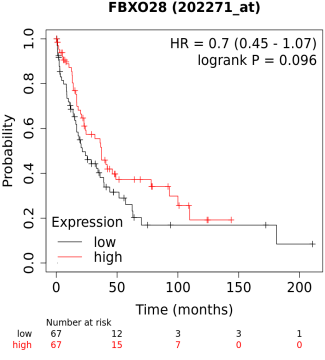

HER2

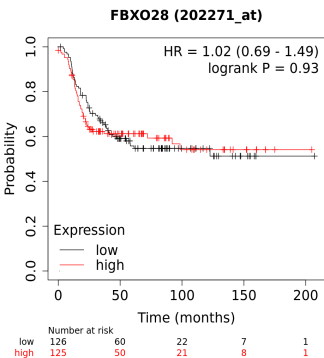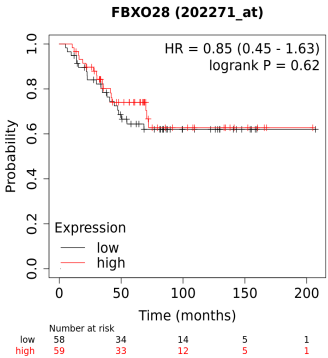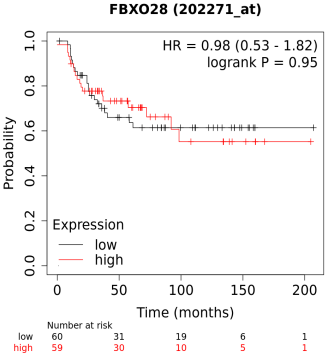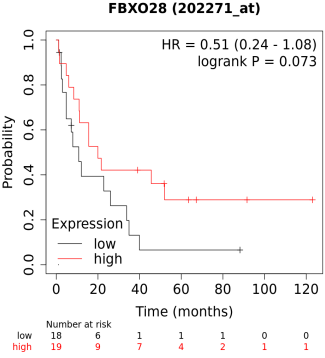

TNBC

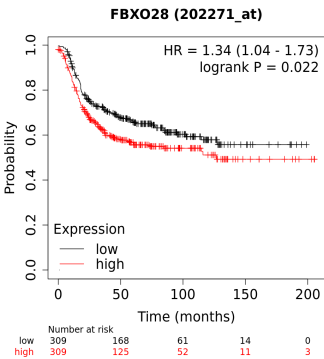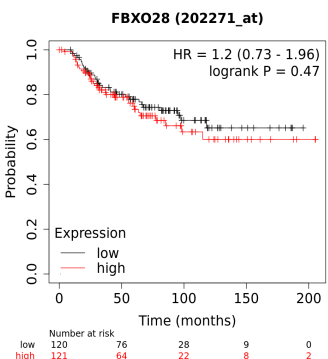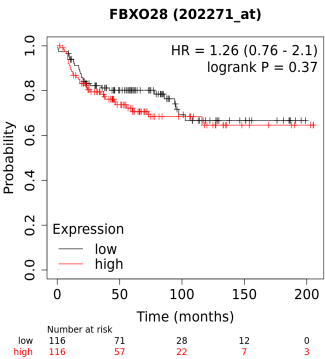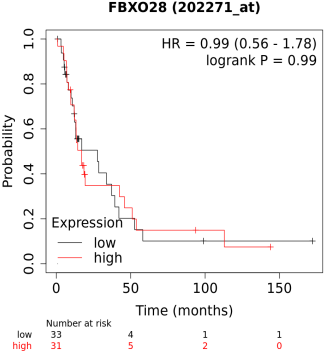

Supplement: Supplementary file 3 — Additional file 3: Figure S3. The prognostic values of FXBO family members in different subtypes of BC patients. The survival curves comparing BC patients with high (red) and low (black) FBXO expression levels were plotted using the Kaplan-Meier Plotter. DFS, disease-free survival rate; OS, the overall survival rate; DMFS, distance metastasis free survival; PPS, post progression survival; The threshold P-value is less-than 0.05. [file 12935_2021_1833_MOESM3_ESM.zip › Figure S3-8.pdf]

# FBXO31

RFS

OS

DMFS

PPS

Luminal A

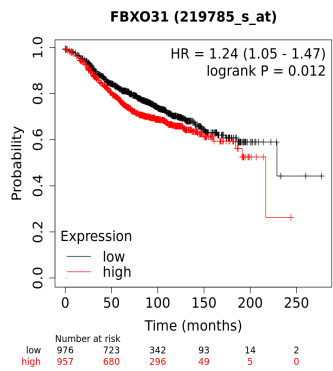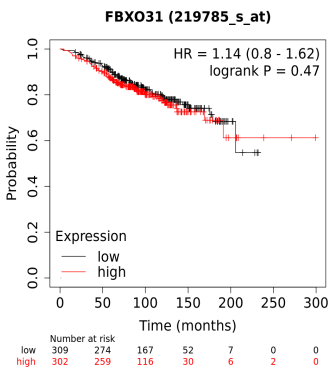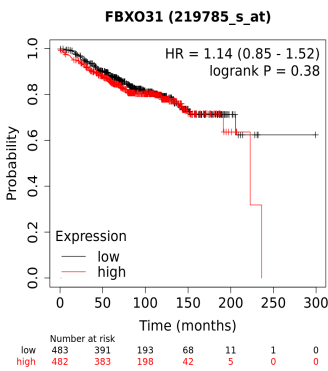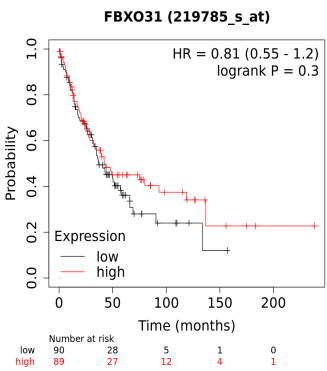

Luminal B

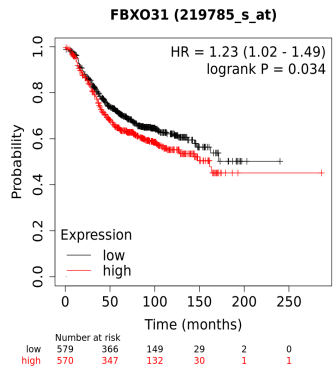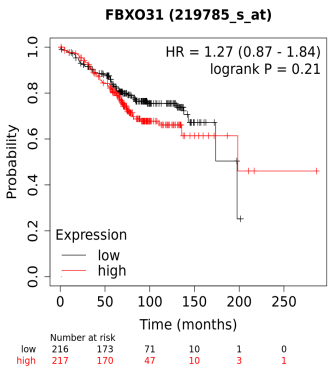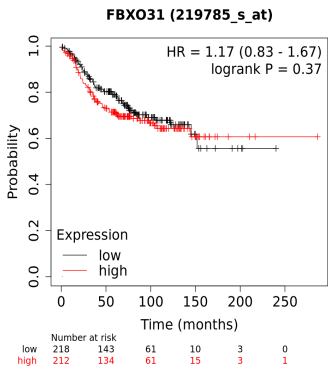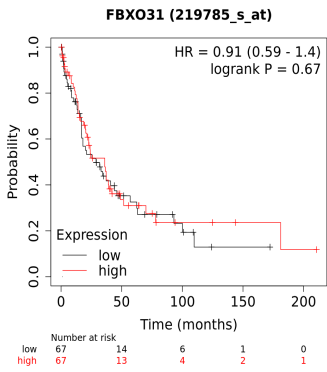

HER2

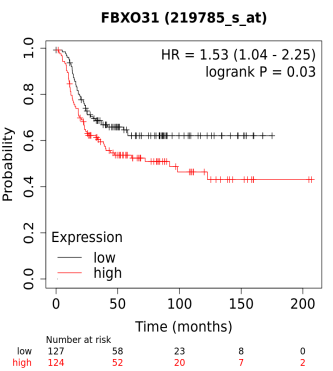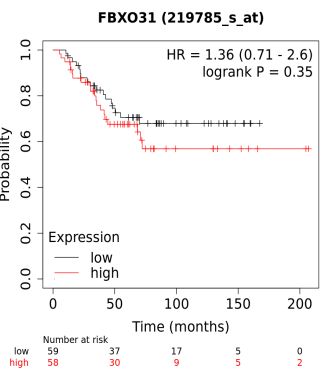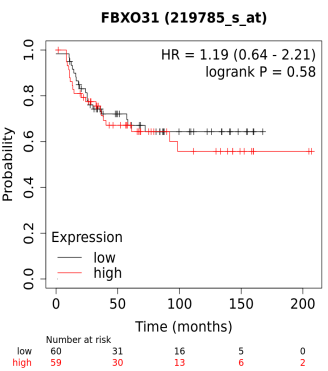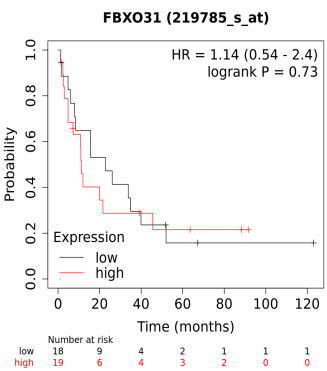

TNBC

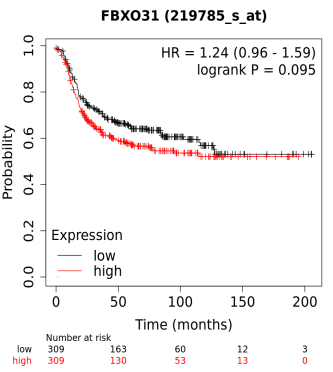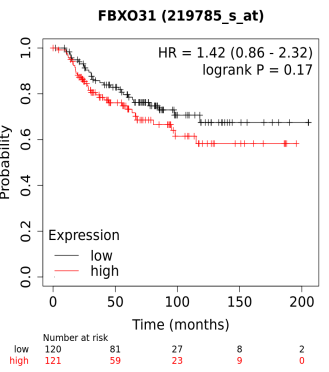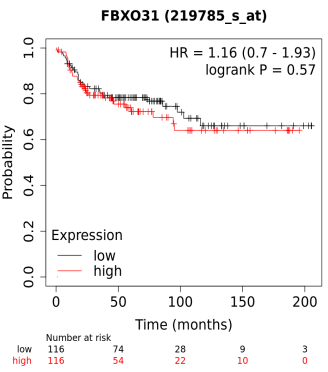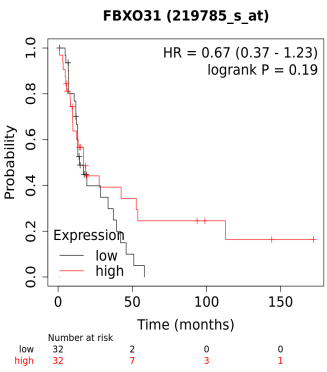

Supplement: Supplementary file 3 — Additional file 3: Figure S3. The prognostic values of FXBO family members in different subtypes of BC patients. The survival curves comparing BC patients with high (red) and low (black) FBXO expression levels were plotted using the Kaplan-Meier Plotter. DFS, disease-free survival rate; OS, the overall survival rate; DMFS, distance metastasis free survival; PPS, post progression survival; The threshold P-value is less-than 0.05. [file 12935_2021_1833_MOESM3_ESM.zip › Figure S3-9.pdf]
