# Supplementary material for: Systematic analysis of the expression and prognosis relevance of FBXO family reveals the significance of FBXO1 in human breast cancer
Source: Cancer Cell Int. 2021 Feb 23;21:130. doi: 10.1186/s12935-021-01833-y (PMC7903729; doi:10.1186/s12935-021-01833-y)
Supplement: Supplementary file 3 — Additional file 3: Figure S3. The prognostic values of FXBO family members in different subtypes of BC patients. The survival curves comparing BC patients with high (red) and low (black) FBXO expression levels were plotted using the Kaplan-Meier Plotter. DFS, disease-free survival rate; OS, the overall survival rate; DMFS, distance metastasis free survival; PPS, post progression survival; The threshold P-value is less-than 0.05. [file 12935_2021_1833_MOESM3_ESM.zip › Figure S3-7.pdf]

FBXO22

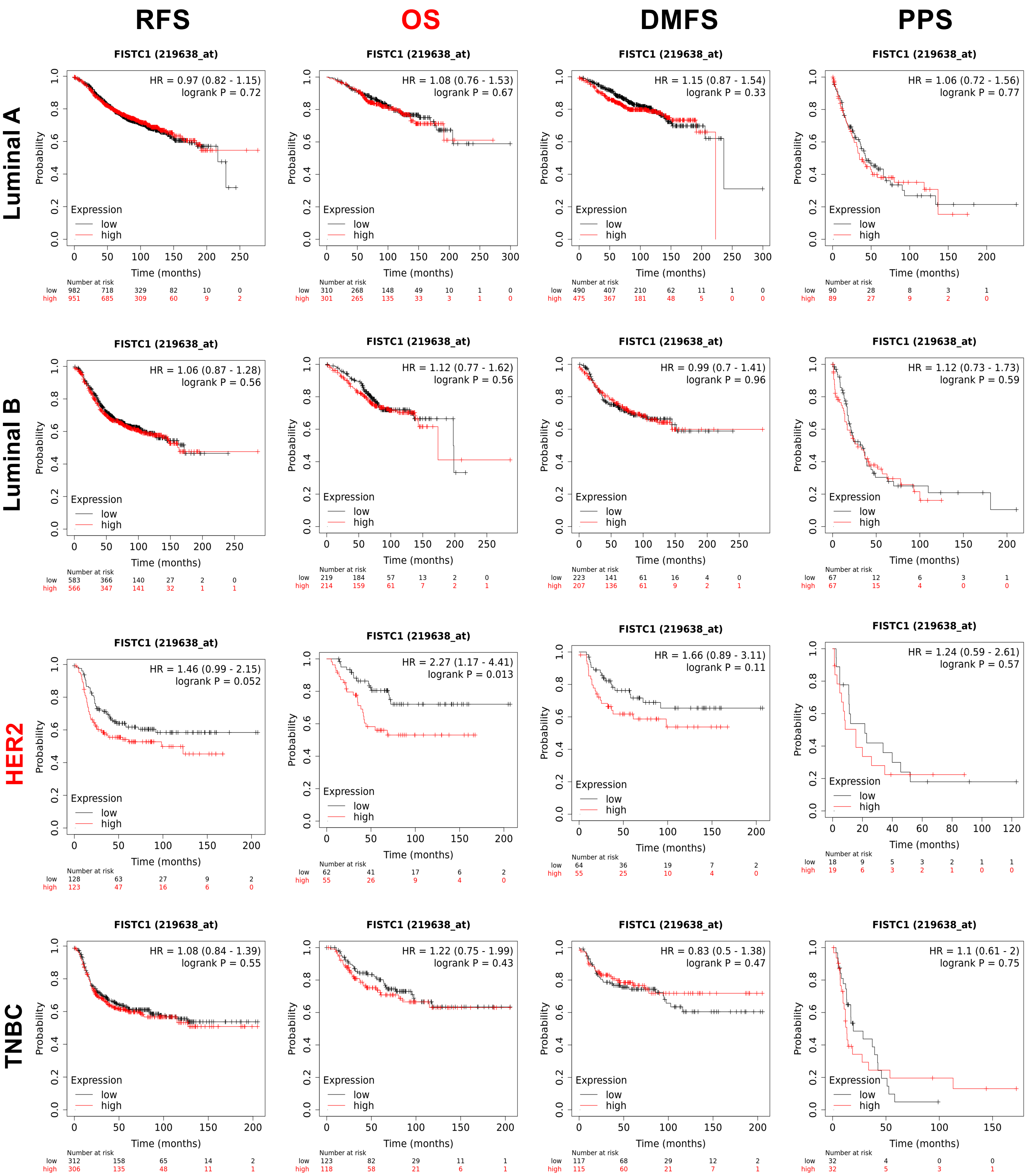
RFS
OS
DMFS
PPS
Luminal A
Luminal B
HER2
TNBC
FISTC1 (219638_at)
HR = 0.97 (0.82 - 1.15)
logrank P = 0.72
HR = 1.08 (0.76 - 1.53)
logrank P = 0.67
HR = 1.15 (0.87 - 1.54)
logrank P = 0.33
HR = 1.06 (0.72 - 1.56)
logrank P = 0.77
HR = 1.06 (0.87 - 1.28)
logrank P = 0.56
HR = 1.12 (0.77 - 1.62)
logrank P = 0.56
HR = 0.99 (0.7 - 1.41)
logrank P = 0.96
HR = 1.12 (0.73 - 1.73)
logrank P = 0.59
HR = 1.46 (0.99 - 2.15)
logrank P = 0.052
HR = 2.27 (1.17 - 4.41)
logrank P = 0.013
HR = 1.66 (0.89 - 3.11)
logrank P = 0.11
HR = 1.24 (0.59 - 2.61)
logrank P = 0.57
HR = 1.08 (0.84 - 1.39)
logrank P = 0.55
HR = 1.22 (0.75 - 1.99)
logrank P = 0.43
HR = 0.83 (0.5 - 1.38)
logrank P = 0.47
HR = 1.1 (0.61 - 2)
logrank P = 0.75
Expression
low
high
Probability
Time (months)
Number at risk
