# Supplementary material for: Systematic analysis of the expression and prognosis relevance of FBXO family reveals the significance of FBXO1 in human breast cancer
Source: Cancer Cell Int. 2021 Feb 23;21:130. doi: 10.1186/s12935-021-01833-y (PMC7903729; doi:10.1186/s12935-021-01833-y)
Supplement: Supplementary file 4 — Additional file 4: Table S1. Summary of clinical data of breast cancer patients whose samples were used in Immunohistochemical Staining. [file 12935_2021_1833_MOESM4_ESM.docx]

**Table S1. Summary of clinical data of breast cancer patients whose samples were used in Immunohistochemical Staining.**

|  | **Molecular subtype** | **Age** | **Gender** | **Pathology** | **ER**  **Statue** | **PR**  **Statue** | **HER2**  **Statue** | **Ki67**  **Statue** | **Histological Grading** | **Vascular/Nerve invasion** | **pTNM**  **T** | **pTNM**  **N** | **pTNM**  **M** | **Stage** |  |
| --- | --- | --- | --- | --- | --- | --- | --- | --- | --- | --- | --- | --- | --- | --- | --- |
| Sample 1 | Luminal A | 37 | Female | Nonspecific invasive carcinoma | 90%  + | 80%  + | - | 15%  + | 2 | V（+）  N（-） | 2 | 0 | 0 | IIa |  |
| Sample 2 | Luminal B | 51 | Female | Nonspecific invasive carcinoma | 80%  + | 1%  - | - | 70%  + | 2 | V（+）  N（+） | 2 | 1 | 0 | IIb |  |
| Sample 3 | HER2+ | 56 | Female | Nonspecific invasive carcinoma | 40%  + | 1%  + | 3+ | 10%  + | 2 | V（-）  N（-） | 1 | 1 | 0 | IIa |  |
| Sample 4 | TNBC | 72 | Female | Nonspecific invasive carcinoma | - | - | - | 50%  + | 3 | V（+）  N（-） | 2 | 0 | 0 | IIa |  |
