# Supplementary material for: Systematic analysis of the expression and prognosis relevance of FBXO family reveals the significance of FBXO1 in human breast cancer
Source: Cancer Cell Int. 2021 Feb 23;21:130. doi: 10.1186/s12935-021-01833-y (PMC7903729; doi:10.1186/s12935-021-01833-y)
Supplement: Supplementary file 5 — Additional file 5: Figure S4. KEGG analysis of cell cycle pathway regulated by the FBXO1 and co-expression genes alteration in BC are shown in DAVID database. Altered genes of the pathway marked in red. [file 12935_2021_1833_MOESM5_ESM.pdf]

Figure S4

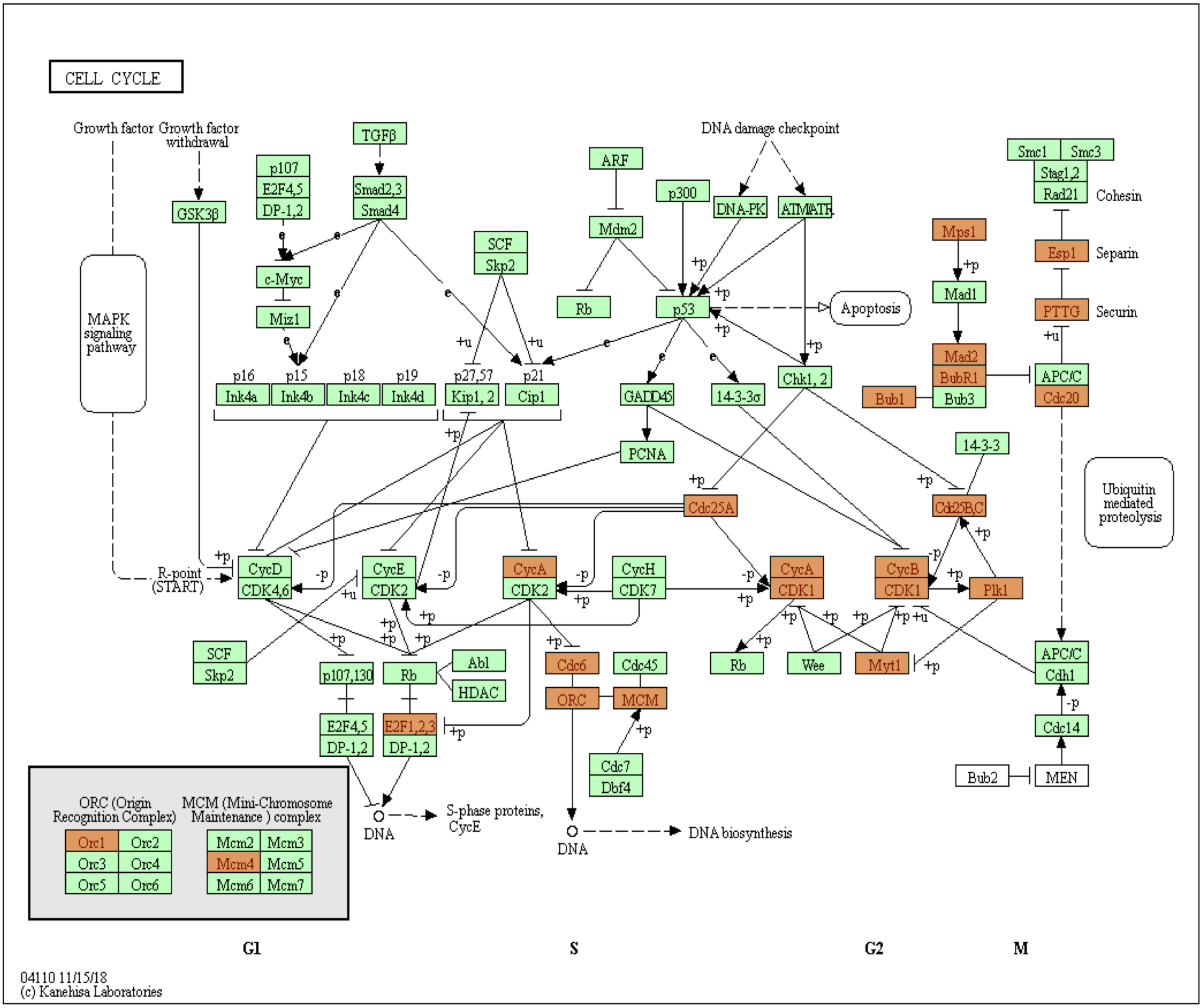
CELL CYCLE
Growth factor
Growth factor withdrawal
MAPK signaling pathway
GSK3β
TGFβ
Smad2,3
Smad4
p107
E2F4,5
DP-1,2
c-Myc
Miz1
SCF
Skp2
ARF
Mdm2
Rb
p300
DNA damage checkpoint
DNA-PK
ATM/ATR
p53
Apoptosis
Chk1, 2
GADD45
14-3-3σ
PCNA
p16
p15
p18
p19
Ink4a
Ink4b
Ink4c
Ink4d
p27,57
Kip1, 2
p21
Cip1
R-point (START)
CycD
CDK4,6
CycE
CDK2
CycA
CDK2
CycH
CDK7
Cdc25A
CycA
CDK1
CycB
CDK1
Plk1
Cdc25B,C
14-3-3
Mps1
Mad1
Mad2
BubR1
Bub3
Bub1
Smc1
Smc3
Stag1,2
Rad21
Cohesin
Esp1
Separin
PTTG
Securin
APC/C
Cdc20
Ubiquitin mediated proteolysis
APC/C
Cdh1
Cdc14
Bub2
MEN
Rb
Wee
Myt1
p107,130
Rb
Abl
HDAC
E2F4,5
DP-1,2
E2F1,2,3
DP-1,2
Cdc6
Cdc45
ORC
MCM
Cdc7
Dbf4
DNA
S-phase proteins, CycE
DNA
DNA biosynthesis
ORC (Origin Recognition Complex)
Orc1
Orc2
Orc3
Orc4
Orc5
Orc6
MCM (Mini-Chromosome Maintenance) complex
Mcm2
Mcm3
Mcm4
Mcm5
Mcm6
Mcm7
G1
S
G2
M
04110 11/15/18
(c) Kanehisa Laboratories
